# Immune Plasma Algorithm: A Novel Meta-Heuristic for Optimization Problems

**DOI:** 10.1109/ACCESS.2020.3043174

**Published:** 2020-12-07

**Authors:** Selcuk Aslan, Sercan Demirci

**Affiliations:** 1 Department of Computer EngineeringNevşehir Hacı Bektaş Veli University 50300 Nevşehir Turkey; 2 Department of Computer EngineeringOndokuz Mayıs University 37139 55200 Samsun Turkey

**Keywords:** Meta-heuristics, immune plasma algorithm, plasma treatment

## Abstract

The recent global health crisis also known as the COVID-19 or coronavirus pandemic has attracted the researchers’ attentions to a treatment approach called immune plasma or convalescent plasma once more again. The main idea lying behind the immune plasma treatment is transferring the antibody rich part of the blood taken from the patients who are recovered previously to the critical individuals and its efficiency has been proven by successfully using against great influenza of 1918, H1N1 flu, MERS, SARS and Ebola. In this study, we modeled the mentioned treatment approach and introduced a new meta-heuristic called Immune Plasma (IP) algorithm. The performance of the IP algorithm was investigated in detail and then compared with some of the classical and state-of-art meta-heuristics by solving a set of numerical benchmark problems. Moreover, the capabilities of the IP algorithm were also analyzed over complex engineering optimization problems related with the noise minimization of the electro-encephalography signal measurements. The results of the experimental studies showed that the IP algorithm is capable of obtaining better solutions for the vast majority of the test problems compared to other commonly used meta-heuristic algorithms.

## I. Introduction

Real world problems or complex engineering designs usually require finding optimum values for hundreds or thousands of decision parameters. Even though there are special analytic methods for solving some of the mentioned optimization problems, they generally utilize from the gradient-derivative based calculations for determining the search direction and qualities of the final solutions change dramatically with chosen start point or points [1], [2]. In order to outcome the possible drawbacks stemmed from the existing work-flow of the classical methods, researchers focused on new problem solving techniques and meta-heuristic algorithms were proposed as an alternative to them [3]–[5].

Each meta-heuristic algorithm tries to model a well-known natural phenomena and applies its model for solving the optimization problems. By considering the natural phenomena that inspire researchers, meta-heuristics can be roughly classified into four groups named evolutionary algorithms, swarm-intelligence based algorithms, physic-based algorithms and human-based algorithms. Evolutionary algorithms are based on Darwin’s theory of survival of the fittest and model common evolutionary mechanisms such as mutation, crossover and selection. Genetic algorithm (GA) proposed by Holland is one of the most popular evolutionary algorithms [6]. Similar to GA, Differential Evolution (DE) algorithm [7], Evolutionary Strategies (ES) [8], Biogeography-Based Optimizer (BBO) [9] also model the well-known evolutionary mechanisms and they are other evolutionary algorithms.

Swarm-intelligence based meta-heuristics try to model intelligent behaviours of the social creatures such as birds, bats, cats, whales and some insects such as ants, bees, moths and grasshoppers. One of the most popular swarm-intelligence based meta-heuristics is Ant Colony Optimization (ACO) algorithm [10]. As its name implies, ACO algorithm is related with the ants and uses source finding and communication capabilities of them. Another important swarm-intelligence based algorithm was proposed by Eberhart and Kennedy and named as Particle Swarm Optimization (PSO) algorithm [11]. The PSO algorithm basically models the movements of the fish schooling and bird flocking. Yang and Deb proposed Cuckoo Search (CS) algorithm by guiding brood reproductive behaviours of cuckoo birds [12]. Yang also introduced a new swarm-intelligence based meta-heuristic called Firefly algorithm for short FA [13]. FA mimics the flashing property of the fireflies used to manage communication. In another study, Yang investigated the advanced echolocation capability of bats that are only mammals with wings and Bat algorithm (BA) was developed [14]. A recent meta-heuristic proposed by Yang was called as Flower Pollination algorithm (FPA) or simply Flower algorithm that mimics the self-pollination and cross-pollination of flowers [15]. Experimental studies showed that FPA is more efficient than PSO and GA [15]. The foraging and communication characteristics of the honey bees become the source of inspiration for Karaboga and Artificial Bee Colony (ABC) algorithm was introduced [16]. Mirjalili *et al.* analyzed the leadership hierarchy and hunting behaviours of a special type of grey wolves and presented Grey Wolf Optimizer (GWO) algorithm [17]. The flying nature of the moths in night and navigation method of them were guided by Mirjalili and Moth-Flame Optimisation (MFO) algorithm was developed [18]. The meta-heuristic techniques introduced by Mirjalili are not limited with the GWO and MFO algorithms. Mirjalili has recently introduced Ant Lion Optimizer (ALO) algorithm that references complex hunting strategies of the antlions [19], Dragonfly algorithm (DA) that mimics the static and dynamic swarming behaviours of the dragonflies [20], Sine Cosine algorithm (SCA) using a mathematical model based on sine and cosine functions [21]. Mirjalili also directly contributed to the development of the Whale Optimization algorithm (WOA) [22], Multi-Verse Optimizer (MVO) [23], Grasshopper Optimisation algorithm (GOA) [24], Salp Swarm algorithm (SSA) [25], Harris Hawks Optimizer (HHO) algorithm [26], Marine Predator algorithm (MPA) [27] and Slime Mould algorithm (SMA) [28]. Chou *et al.* modeled the hunting, learning and terriority determining characteristics of the jaguars and proposed Jaguar algorithm for short JA [29]. The social relationship and collaborative behavior of the spotted hyenas gave inspiration to Dhiman and Kumar and they introduced Spotted Hyena Optimizer (SHO) [30]. Dhiman and Kumar also proposed Seagull Optimization algorithm (SOA) by guiding migration and attacking behaviors of a seagull [31]. Border Collie is one of the most smartest breeds of dogs and has unique herding style. By referencing the sheep herding styles of Border Collie dogs, Dutta *et al.* introduced Border Collie Optimization (BCO) algorithm [32].

The third group of the meta-heuristics rely on some of the well-known physical laws or the mechanisms that start and manage complex chemical reactions. Electromagnetism-like algorithm (EMA) inspired by the fundamental electromagnetism was introduced by Birbil and Fang [33]. The law of gravity was utilized by Rashedi *et al.* and Gravitational Search algorithm (GSA) was presented [34]. Central Force Optimization (CFO) was proposed by Formato with the guidance of gravitational kinematics [35]. Shah-Hosseini suggested Intelligent Water Drops (IWD) algorithm [36]. In IWD algorithm, movement of a water drop from one point of the river to another was referenced while searching the solutions of the problem [36]. Refraction and reflection of light rays were modeled in the Light Ray Optimization (LRO) [37]. Snell’s law that describes the relationship between the angles of the incident and reflected rays were referenced in the Ray Optimization (RO) algorithm [37]. Cuevas *et al.* used physical principles of the thermal-energy motion mechanism and proposed States of Matter Search (SMS) algorithm [38]. The nuclear collision reactions including scattering and absorption were used by Wagner *et al.* and Particle Collision algorithm (PCA) was proposed [39]. The push and pull forces of positive and negative ions gave inspiration to Javidy *et al.* and Ions Motion algorithm (IMO) was introduced [40]. The final group of meta-heuristics imitates the human behaviours or operations to do with the being human. Tabu Search (TS) algorithm by Glover is one of the most famous human-based meta-heuristics [41]. It was designed to prevent the search mechanics from the local minimas stored in a tabu list or memory. Kumar *et al.* introduced Socio Evolution Learning Optimization (SELO) algorithm by analyzing how humans organized as families effect other individuals and trigger a social learning process [42]. The teaching and learning order in a classroom was investigated by Rao *et al.* and Teaching Learning Based Optimization (TLBO) algorithm was proposed [43].

When the short story of the meta-heuristics given above is investigated, it might be thought that existing algorithms are enough and there is no need for a new meta-heuristic technique. However, No-Free-Lunch (NFL) theorem states that each meta-heuristic algorithm has different capabilities and a single algorithm for solving all optimization problems with the highest efficiency does not exist [5]. As an expected result of this situation, designing new meta-heuristic algorithm after analyzing work-flow of an intelligent organizations of nature still protects its importance for further advances in computer, information and other engineering disciplines. In this study, a new meta-heuristic algorithm named as Immune Plasma (IP) algorithm was introduced. The IP algorithm is the first meta-heuristic technique that is based on a treatment method known as immune plasma or convalescent plasma. Experimental studies carried out with a set of different optimization problems showed that IP algorithm is capable of obtaining better solutions for most of the problems compared to the other meta-heuristics. The rest of this article is organized as follows: The background of the immune plasma technique and how its fundamental operations are guided for describing steps of the IP algorithm are given in Section II. The results of the experiments and comparative studies are presented in Section III. Finally, conclusion and possible future works with the IP algorithm are summarized in Section IV.

## II. Immune Plasma Algorithm

**FIGURE 1.**
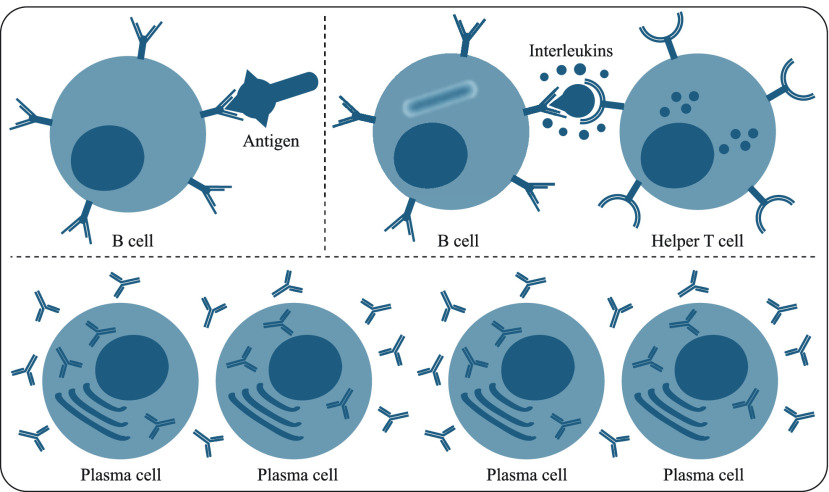
The relationship between B, T and plasma cells.

An antigen can be described as a foreign invader such as parasite, fungi, bacteria or virus or a part of these creatures that can cause an infection in the host [44]–[46]. When an infection is triggered by the related antigens, the immune system starts a set of complex defence procedures to seek out and destroy them with the help of the lymphoid organs that are responsible for producing and maturing lymphocytes such as T and B lymphocytes [44]–[46]. The T and B lymphocytes or cells play key roles in the adaptive immune response of the whole system and immune memory of the host. Both T and B lymphocytes are produced in the bone marrow. However, while the T cells mature in the thymus and T stands for thymus, the B cells continue maturing in the bone marrows [44]–[46]. The B cells are equipped with B cell receptors on their membrane and can bind to specific antigen with these receptors [44]–[46]. When the B lymphocyte binds to a specific antigen, it calls upon a kind of T cells named as helper T cell or Th cell. The helper T cell secretes chemicals known as interleukins and interferons near to the B lymphocytes. These secretions allow the B lymphocytes to multiply. Moreover, they mature the B cells into plasma cells [44]–[46]. The Fig. 1 illustrates how a B cell binds to a specific antigen and matures into plasma cell with the help of Th cell.

**FIGURE 2.**
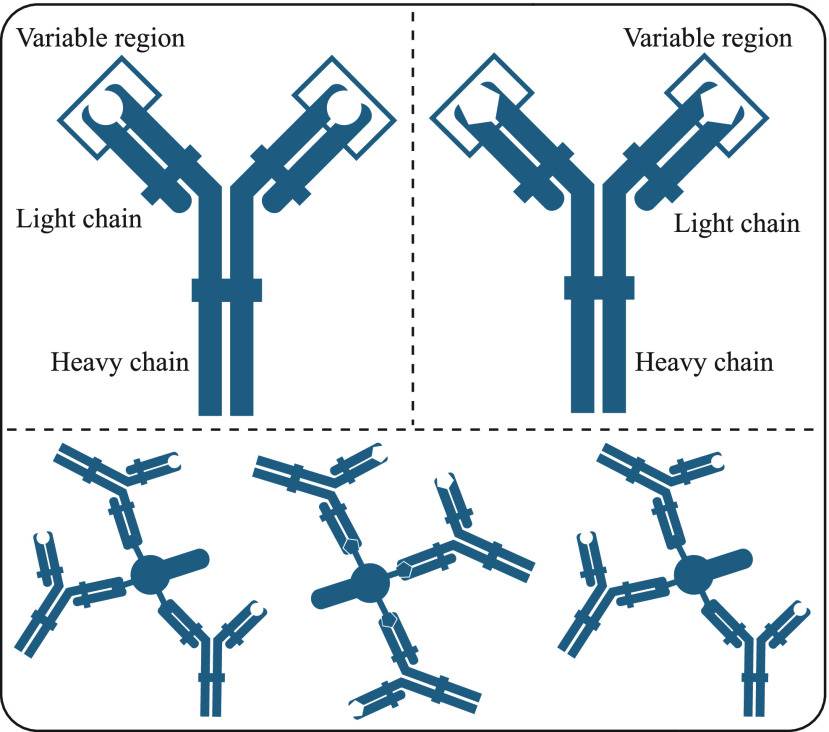
Structure of an antibody and connection with an antigen.

Plasma cells contribute to the defence by producing antibodies that are also called immunoglobulins for short Igs [44]–[46]. Antibodies are actually proteins and each antibody consists of two heavy and two light polypeptide chains. The heavy and light chains connect via covalent bonds and they form a Y-shaped structure [44]–[46]. The chains of an antibody contain constant and variable regions. The constant regions are responsible for binding other structures such as the membranes of the different immune system cells. The variable regions are designed with a specific set of amino acids and help to bind particular antigens for which the antibodies are build [44]–[46]. The constant and variable regions of two hypothetical antibodies and how they bind to their specific antigens are depicted in the Fig. 2.

Each plasma cell is programmed to synthesize one specific antibody and antibodies visible in the blood or seen on the membranes of the immune system cells. In either existence form, they contribute the defence mechanism for the several scenarios given below:

• When a free-floating antibody determines and binds to its particular antigen, a virus can lose its capabilities that manage the virus binding operations on the receptors of the healthy cells.
• The antibodies can label particular antigens or infected cells for the other immune system cells that can destroy them.
• The antibodies and their particular antigens can be aggregated to form insoluble antibody-antigen complex and inactivate the functionalities of pathogens.

**FIGURE 3.**
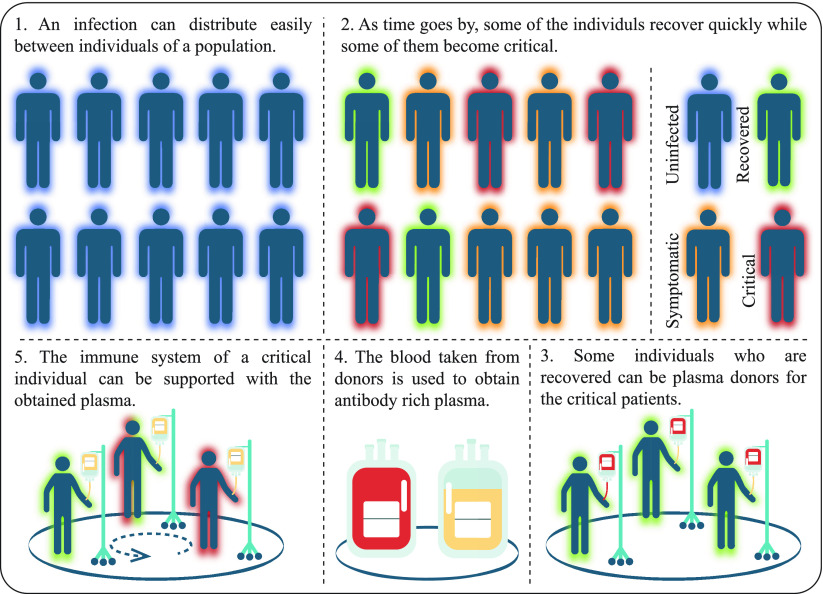
General concept of the IP algorithm.

The antibody response of the immune system to an antigen never seen before is given within a few weeks [44]–[46]. After an infection begins, the level of specific antibodies rises slowly and reaches to a peak around ten days and decreases with time. However, the level of these specific antibodies is not low as in the initial state of the infection and the immune system produces a huge volume of higher avidity antigens between one or three days for the subsequent challenges with the same antigen [44]–[46]. As stated earlier, the plasma cells produce antibodies and secrete them into the bloodstream. However, some individuals with the weak immune systems or immune system diseases are not capable of producing sufficient amounts of antibodies [44]–[46]. For the infected individuals, the blood or antigen rich part of it called plasma from patients who have recovered for the same infection can be used as a valuable source and a treatment method known as the convalescent plasma or immune plasma has been introduced and tested successfully for a wide variety of diseases [47]–[49]. The strong and biologically evident ideas lying behind the immune plasma treatment guide designing a completely new meta-heuristic named as IP algorithm. In the IP algorithm, while each individual in the population indicates a possible solution of the problem being optimized, immune response of an individual represents the quality of the corresponding solution. The defence operations managed by the immune system for maturing the B cells and producing specific antibodies of the antigen at the beginning of the infection directly contribute to the exploration or diversification characteristic of the IP algorithm. Moreover, determining the individual or individuals recovered shortly after from an infection and transferring the plasma or plasmas to the critical individual or individuals with the same infection maintain a steady exploitation or intensification in the IP algorithm. For understanding the general concept of the IP algorithm, The Fig. 3 should be investigated.

### A. Generating Initial Individuals

As stated earlier, each individual of the IP algorithm corresponds to the possible solution of the problem being considered. Assume that there are $D$ different decision parameters of the considered optimization problem, the $kth$ individual of the population of size $PS$ showed by the $x_{k}$ can be generated with the Eq. (1). In Eq. (1), $x_{kj}$ is matched with the *jth* decision parameter of the $x_{k}$. Also, $x_{j}^{low}$ and $x_{j}^{high}$ are lower and upper bounds of the *jth* parameter, respectively. Finally, $rand(0,1)$ is a randomly determined number between 0 and +1.

\begin{align*}&\hspace {-.5pc} x_{kj} = x_{j}^{low} + rand(0,1)(x_{j}^{high}-x_{j}^{low}) \\&\qquad\qquad \displaystyle { k=\{1,2, \ldots,PS\} \: and \: j = \{1,2,\ldots,D\} } \tag{1}\end{align*}

### B. Infection Spreading and Immune System Response

A small portion of infected individuals can effect the whole population. The secretions of the infectious individuals carry the antigens. When the secretions of an infectious individual enter the body of another individual, the immune system of the new host gives a specific response to protect the host against an infection. For modeling how the secretions of an individual suffering from a possible infection effects another individual and trigger the immune system to generate a specific response, the Eq. (2) given below is used by the IP algorithm. In Eq. (2), $x_{kj}$ is the randomly determined *jth* parameter of the $x_{k}$ individual being infected and $x_{kj}^{inf}$ is the newly calculated *jth* parameter of the $x_{k}^{inf}$ that is used on behalf of the infectious $x_{k}$ individual. The $x_{mj}$ is also the *jth* parameter of the previously infected and randomly selected $x_{m}$ individual.

\begin{align*}&\hspace {-.5pc} x_{kj}^{inf} = x_{kj} + rand(-1,+1)(x_{kj} - x_{mj}) \\&\quad \displaystyle { k=\{1,2,\ldots,PS\} \: and \: m = \{1,2, \ldots,PS\}-\{k\} } \tag{2}\end{align*}

If the immune system response of the infectious $x_{k}$ individual is initiated quickly and the amount of specialized antibodies is increased substantially, it is said that the immune system of the $x_{k}$ individual recognizes the antigens and immune system memory is updated for the subsequent encounter of the same pathogens-antigens or their mutants. In IP algorithm, the amount of specific antibodies of an individual is directly related with the objective function value calculated for the same individual. Assume that $f$ is the objective function of the optimization problem being considered and $x_{k}$ is the *kth* individual of the population of size $PS$. When the $x_{k}^{inf}$ and its objective function value showed by $f(x_{k}^{inf})$ is better than the objective function value of the $x_{k}$ showed by $f(x_{k})$, the *jth* parameter of the $x_{k}$ is set to $x_{kj}^{inf}$. Otherwise, the *jth* parameter of the $x_{k}$ individual remains without change as in the Eq. (3) for a minimization problem.

\begin{align*} x_{kj}=\begin{Bmatrix} x_{kj}^{inf}; &\quad if\:\:f(x_{kj}^{inf}) < f(x_{k})\\ x_{kj}; &\quad if\:\:f(x_{kj}^{inf}) \geq f(x_{k}) \end{Bmatrix}\tag{3}\end{align*}

### C. Plasma Extraction and Transfer

The immune system responses of the individuals to an infection can be vary in one individual to another. While some of the infected individuals require intense care, some of the individuals in the same infected population recover more quickly without complex treatments and they can contribute to the treatment processes of the emerging patient or patients. One of the ways in which recovered individuals contribute to the treatment of the emerging patients is the method known as the convalescent plasma or immune plasma. The immune plasma method mainly depends on transferring antibodies from the blood of the recovered individuals into the critical patients. In the IP algorithm, plasma transfer operations are started by determining number of donors ($NoD$) and number of receivers ($NoR$). After determining $NoD$ and $NoR$, IP algorithm decides which individuals should be donors and which individuals should be receivers. While the donor individuals are determined as the best $NoD$ individual or individuals of the populations of size $PS$, the receiver individuals are determined as the worst $NoR$ individual or individuals in the same population. Assume that $x_{k}^{rcv}$ is the *kth* receiver individual and $x_{m}^{dnr}$ is the randomly determined donor individual. For modeling the plasma transfer from the $x_{m}^{dnr}$ individual to the $x_{k}^{rcv}$ receiver individual, the IP algorithm uses the Eq. (4) given below.

\begin{align*}&\hspace {-.5pc}x_{kj}^{rcv-p} = x_{kj}^{rcv} + rand(-1,+1)(x_{kj}^{rcv} - x_{mj}^{dnr}) \\&\qquad\qquad\qquad\qquad\qquad\qquad\qquad \displaystyle {j = \{1,2,\ldots,D\}} \tag{4}\end{align*}

In Eq. (4), $x_{k}^{rcv-p}$ individual represents the $x_{k}^{rcv}$ after plasma treatment and $x_{kj}^{rcv-p}$ is matched with the *jth* parameter of the $x_{k}^{rcv-p}$ individual. If the immune system response of the $x_{k}^{rcv}$ individual after one dose plasma showed by $f(x_{k}^{rcv-p})$ is better than the immune system response of the donor individual showed by $f(x_{m}^{dnr})$, the $x_{k}^{rcv}$ individual is updated with the $x_{k}^{rcv-p}$ and plasma treatment for $x_{k}^{rcv}$ continues with the $x_{m}^{dnr}$ donor individual. When the decision mechanism that is used to update $x_{k}^{rcv}$ with $x_{k}^{rcv-p}$ is investigated, it is clearly seen that $x_{k}^{rcv-p}$ must be better than $x_{m}^{dnr}$ and intrinsically better than $x_{k}^{rcv}$. The idea lying behind executing this kind of comparative mechanism is to provide a clear vision about the efficiency of the plasma treatment for the $x_{k}^{rcv}$ and possible usage of the next dose of plasma. If the first dose of the plasma significantly contributes to the $x_{k}^{rcv}$ and immune response of the $x_{k}^{rcv}$ after plasma treatment becomes better than the immune response of the $x_{m}^{dnr}$ donor individual, it is assumed that the $x_{k}^{rcv}$ receiver is now capable of resisting the infection similar to the $x_{m}^{dnr}$ donor and the plasma treatment should be continued. For the subsequent plasma dose, if the immune response of the $x_{k}^{rcv}$ individual after new dose showed by $f(x_{k}^{rcv-p})$ is better than the immune system response of the receiver individual showed by $f(x_{k}^{rcv})$, the $x_{k}^{rcv}$ individual is updated with the $x_{k}^{rcv-p}$ and plasma treatment of $x_{k}^{rcv}$ continues with the $x_{m}^{dnr}$ donor individual. Because of the reason that new $x_{k}^{rcv}$ is already better than the $x_{m}^{dnr}$ if the first dose of the plasma successes, the decision whether the plasma treatment continues with the next dose or not is made by simply comparing $f(x_{k}^{rcv})$ and $f(x_{k}^{rcv-p})$ values.

The plasma treatment should be completed at a time after controlling its efficiency on the selected patient. If the immune system response of the $x_{k}^{rcv}$ for the first dose of plasma is not better than the immune system response of the $x_{m}^{dnr}$, the $x_{k}^{rcv}$ receiver is simply strengthen by changing its parameters with the corresponding parameters of the $x_{m}^{dnr}$ donor individual to guarantee that one dose plasma is transferred and then plasma treatment is completed. If it is understood that the plasma treatment will be completed after the second or subsequent doses, there is no need to update the $x_{k}^{rcv}$ receiver with the information of the $x_{m}^{dnr}$ because of the contribution of the first plasma dose on the $x_{k}^{rcv}$. The Alg. (1) describes in detail how the mentioned treatment strategy is executed in the IP algorithm.

Algorithm 1 **Plasma Transfer Operations in the IP Algorithm**
1: //Plasma transfer for critical individuals
2: $doseControl[1 \ldots NoR] \gets $ set each element to 1
3: $dIndexes[1 \ldots NoD] \gets $ get the indexes of the donors
4: $rIndexes[1 \ldots NoR] \gets $ get the indexes of the receivers
5: $treatmentControl[1 \ldots NoR] \gets $ set each element to 1
6: **for** $i \gets 1 {\dots } NoR$ **do**
7: $k \gets rIndexes[i]$ and $m \gets $ a random element from $dIndexes$
8: $x_{k}^{rcv}, x_{m}^{dnr} \gets $ get the $kth$ and $mth$ individuals from the population
9: **while** $treatmentControl[i] == 1 $ **do**
10: **if** $t_{cr} < t_{max}$ **then**
11: $t_{cr} \gets t_{cr} + 1$ and $x_{k}^{rcv-p} \gets $ plasma treatment to $x_{k}^{rcv}$ with $x_{m}^{dnr}$ using Eq. (4)
12: **if** $doseControl[i] == 1 $ **then**
13: **if** $f(x_{k}^{rcv-p}) < f(x_{m}^{dnr})$ **then**
14: $doseControl[i] \gets doseControl[i] + 1$ and update $x_{k}^{rcv}$ with $x_{k}^{rcv-p}$
15: **else**
16: Update $x_{k}^{rcv}$ with $x_{m}^{dnr}$
17: Set $treatmentControl[i]$ to 0 for completing transfer
18: **end if**
19: **else**
20: **if** $f(x_{k}^{rcv-p}) < f(x_{k}^{rcv})$ **then**
21: Update $x_{k}^{rcv}$ with $x_{k}^{rcv-p}$
22: **else**
23: Set $treatmentControl[i]$ to 0 for completing transfer
24: **end if**
25: **end if**
26: Update $x_{best}$ with $x_{k}^{rcv}$ if $f(x_{k}^{rcv}) < f(x_{best})$
27: **end if**
28: **end while**
29: **end for**

The immune response or antibody level of an individual who recovered and contributed to the treatment processes of the emerging patient or patients as plasma donor changes as time goes by. With the completion of the plasma treatment, IP algorithm applies a controlled-randomize procedure for changing the previously determined donor individual or individuals. If the random number generated between 0 and +1 is less than the ratio between current fitness evaluation ($t_{cr}$) and predetermined maximum fitness evaluations ($t_{max}$), each parameter of the $x_{m}^{dnr}$ individual or solution is modified by guiding the previously assigned values of them as described in Eq. (5). When the approach modeled for updating donor individual or individuals is analyzed, it is clearly seen that the probability of protecting antibody composition of a donor individual is increased with the subsequent evaluations. By executing this kind of mechanism, a donor individual can get a chance of strengthening the immune system memory for the same or similar infections. However, if the random number generated between 0 and +1 is higher than the $t_{cr}/t_{max}$, it is assumed that the immune system response of the individual helped critical patient or patients as donor is strong but not enough to obtain stationary antibody memory for the current antigen and its parameters are re-initialized with the Eq. (1).

\begin{equation*} x_{mj}^{dnr} = x_{mj}^{dnr} + rand(-1,+1)x_{mj}^{dnr}\tag{5}\end{equation*}

For summarizing how IP algorithm generates initial individuals, manages distribution of infection, determines immune responses, selects donor and receiver individuals, uses plasmas for the receivers and finally updates donors, the detailed pseudo-code given in the Alg. (2) should be investigated. The infection distribution (lines between 6 and 14 in the pseudo-code of Alg. (2)) is the stage that maintains the diversification or exploration property of the IP algorithm. As mentioned before, the individuals of the population correspond to the possible solutions of IP algorithm. In the infection distribution, each individual is infected with a randomly selected individual. In other words, a candidate solution is generated at the neighborhood of each individual and problem space gets a chance of searching. The plasma transfer between donor and receiver individuals (lines between 16 and 44 in the pseudo-code of Alg. (2)) helps the exploitation or intensification operations. Especially, when a receiver is treated with the same plasma, the neighborhood of the solution represented with that receiver is searched more sensitively. The donor update (lines between 46 and 57 in the pseudo-code of Alg. (2)) is a stage that contributes both exploitation and exploration properties of the IP algorithm. At this state, a donor can be changed completely and a new region in the search space is discovered. In addition to this, a donor can be modified slightly and the neighborhood of the solution represented with this donor is searched more sensitively.

Algorithm 2 **Fundamental Steps of the IP Algorithm**
1: Assign values to $PS$, $D$, $NoD$ and $NoR$ control parameters.
2: Set $x_{best}$ as the best individual of the $PS$ individuals.
3: Select $t_{max}$ and set $t_{cr}$ to $PS$.
4: **while** $t_{cr} < t_{max}$ **do**
5: //Infection distribution
6: **for** $k \gets 1 {\dots } PS$ **do**
7: **if** $t_{cr} < t_{max}$ **then**
8: $t_{cr} \gets t_{cr} + 1$ and $x_{k}^{inf} \gets $ infect $x_{k}$ with $x_{m}$ using Eq. (2)
9: **if** $f(x_{k}^{inf}) < f(x_{k})$ **then**
10: Update $x_{k}$ with $x_{k}^{inf}$ as described in Eq. (3)
11: Update $x_{best}$ with $x_{k}$ if $f(x_{k}) < f(x_{best})$
12: **end if**
13: **end if**
14: **end for**
15: //Plasma transfer for critical individuals
16: $doseControl[1 \ldots NoR] \gets $ set each element to 1
17: $dIndexes[1 \ldots NoD] \gets $ get the indexes of the donors
18: $rIndexes[1 \ldots NoR] \gets $ get the indexes of the receivers
19: $treatmentControl[1 \ldots NoR] \gets $ set each element to 1
20: **for** $i \gets 1 {\dots } NoR$ **do**
21: $k \gets rIndexes[i]$ and $m \gets $ a random element from $dIndexes$
22: $x_{k}^{rcv}, x_{m}^{dnr} \gets $ get the $kth$ and $mth$ individuals from the population
23: **while** $treatmentControl[i] == 1 $ **do**
24: **if** $t_{cr} < t_{max}$ **then**
25: $t_{cr} \gets t_{cr} + 1$ and $x_{k}^{rcv-p} \gets $ plasma treatment to $x_{k}^{rcv}$ with $x_{m}^{dnr}$ using Eq. (4)
26: **if** $doseControl[i] == 1 $ **then**
27: **if** $f(x_{k}^{rcv-p}) < f(x_{m}^{dnr})$ **then**
28: $doseControl[i] \gets doseControl[i] + 1$
29: Update $x_{k}^{rcv}$ with $x_{k}^{rcv-p}$
30: **else**
31: Update $x_{k}^{rcv}$ with $x_{m}^{dnr}$
32: Set $treatmentControl[i]$ to 0 for completing transfer
33: **end if**
34: **else**
35: **if** $f(x_{k}^{rcv-p}) < f(x_{k}^{rcv})$ **then**
36: Update $x_{k}^{rcv}$ with $x_{k}^{rcv-p}$
37: **else**
38: Set $treatmentControl[i]$ to 0 for completing transfer
39: **end if**
40: **end if**
41: Update $x_{best}$ with $x_{k}^{rcv}$ if $f(x_{k}^{rcv}) < f(x_{best})$
42: **end if**
43: **end while**
44: **end for**
45: //Donor update
46: **for** $i \gets 1 {\dots } NoD$ **do**
47: **if** $t_{cr} < t_{max}$ **then**
48: $t_{cr} \gets t_{cr} + 1$ and $m \gets dIndexes[i]$
49: $x_{m}^{dnr} \gets $ get the $mth$ individual from the population
50: **if** $(t_{cr}/t_{max}) < rand(0,1)$ **then**
51: Update $x_{m}^{dnr}$ using Eq. (5)
52: **else**
53: Update $x_{m}^{dnr}$ using Eq. (1)
54: **end if**
55: Update $x_{best}$ with the $x_{m}^{dnr}$ if $f(x_{m}^{dnr}) < f(x_{best})$
56: **end if**
57: **end for**
58: **end while**

When the detailed pseudo-code of the IP algorithm given in the Alg. (2) is controlled, it is clearly seen that the number of evaluations or number of calls to calculate objective function values per cycle-iteration can vary. If the first dose of the plasma significantly contributes to the treatment of a patient, the subsequent dose is prepared and plasma transfer continues. Even though the number of evaluations per cycle changes in the IP algorithm, it does not cause a problem for the fair comparison with other meta-heuristics. For a fair comparison between meta-heuristics, the value of maximum evaluations abbreviated as $t_{max}$ in the Alg. (2) is determined first and then competitor meta-heuristics are terminated when they reach to the predetermined $t_{max}$. Assuming that the IP algorithm is used for solving a $D$-dimensional problem requiring calculation of an objective function with the complexity of $O(D)$, the running time of the IP algorithm is found equal to $O(t_{max} \times D)$.

The calculation of the running time of the IP algorithm in terms of maximum evaluation numbers and cost of objective function simplifies a generalized comparison with other meta-heuristics. However, the effect of the interior operations carried out in the infection distribution, plasma transfer and donor update should also be taken into account and included in the calculation of the running time of the IP algorithm. When the IP algorithm with the $PS$ individuals is used to solve $D$-dimensional problem of $O(D)$, the computational complexity of the infection distribution is found $O(PS \times D)$. For determining receiver and donor individuals, the IP algorithm utilizes from a sorting algorithm for which its computational complexity is $O(PS \times logPS)$. After determining receiver and donor individuals, the computational complexity of giving one dose plasma for each of the $NoR$ receivers and updating $NoD$ donors is found $O(PS \times logPS + NoR \times D + NoD \times D)$. By considering the special requirements of different operations, the overall computational complexity of the IP algorithm for a cycle or iteration is defined as $O(PS \times logPS + D \times (PS + NoR + NoD))$.

## III. Experimental Studies

In order to analyze the solving capabilities of the IP algorithm on different scenarios, the whole experimental studies were divided into four parts.^1^ In the first part of the experimental studies, IP algorithm was tested on solving 30-dimensional benchmark problems by assigning different values to the $PS$, $NoR$ and $NoD$ control parameters. The results of the IP algorithm were also compared with the results of the PSO [11], DE [7], RCBBO [9], CS [12], FA [13], GSA [34], ABC [16] and AMO [50] algorithms. The second part of the experimental studies was devoted to the performance investigation of the IP algorithm on high-dimensional problems. The benchmark problems including 100 and 200 parameters were solved by the IP algorithm and its results were compared with the PSO [11], GSA [34], BA [14], FPA [15], SMS [38], FA [13], GA [6], MFO [18] and ALO [19] algorithms. In the third part of the experimental studies, ten bound constrained, single-objective and computationally expensive benchmark functions first presented at the CEC 2015 were solved with the IP algorithm. The results of the IP algorithm for CEC 2015 benchmark functions were compared with the results of the SOA [31], SHO [30], GWO [17], PSO [11], MFO [18], MVO [23], SCA [21], GSA [34], GA [6] and DE [7]. Finally, in the fourth part of the experimental studies, a complex engineering problem that depends on decomposing EEG signal into noise and noise-free parts was solved with the IP algorithm and its results were compared with the results of the GA [6], PSO [11], DE [7], ABC [16], GSA [34], MFO [18], SCA [21] and SSA [25].^1^Source code is available upon request.

### A. Solving Classical Benchmark Problems With IP Algorithm

The experimental studies in this subsection is related to the analysis of the IP algorithm over the 30 dimensional classical benchmark functions. Formulations, lower and upper bounds of these functions are summarized in the Table 1. For the benchmark functions given in the Table 1 except the $f_{8}$, the global minimum value is zero. Only for the $f_{8}$ function, the global minimum value is equal to $-D \times 418.98$ where $D$ shows the number of parameters. In the experiments, the population size of the IP algorithm is set to 30. In order to understand that how the performance of the IP algorithm changes with the different values of the $NoR$ and $NoD$ parameters, nine different combinations are used. The total evaluation number is set to 150,000 for the $f_{1}$, $f_{6}$, $f_{10}$, $f_{12}$ and $f_{13}$ functions, 200,000 for the $f_{2}$ and $f_{11}$ functions, 300,000 for the $f_{7}$, $f_{8}$ and $f_{9}$ functions. Finally, the total evaluation number is set to 500,000 for the $f_{3}$, $f_{4}$ and $f_{5}$ functions. For each benchmark function, 30 independent runs with random seeds are carried out. The mean best objective function values and standard deviations are recorded and summarized in the Table 2.

**TABLE 1:** Classical Benchmark Functions Used in Experiments

Name	Range	Formulation
Sphere	[−100, 100]	$f_{1}(\vec {x})=\sum _{i=1}^{D}\left ({x_{i}^{2} }\right)$
Schwefel2.22	[−10, 10]	$f_{2}(\vec {x})=\sum _{i=1}^{D}|x_{i}| + \prod _{i=1}^{D}|x_{i}|$
Schwefel1.2	[−100, 100]	$f_{3}(\vec {x})=\sum _{i=1}^{D}\sum _{j=1}^{i}x_{j}$
Schwefel1.2	[−100, 100]	$f_{4}(\vec {x})=max_{i}(|x_{i}|, \: 1 \leq i \leq D)$
Rosenbrock	[−30, 30]	$f_{5}(\vec {x})=\sum _{i=1}^{D-1} \left ({100(x_{i+1} - x_{i}^{2} }\right)^{2} + \left ({x_{i}-1 }\right)^{2}) $
Step	[−100, 100]	$f_{6}(\vec {x})=\sum _{i=1}^{D}\left ({\lfloor x_{i} + 0.5 \rfloor }\right)^{2}$
Random	[−1.28, 1.28]	$f_{7}(\vec {x})=\sum _{i=1}^{D}\left ({ix_{i}^{4} }\right) + random[0,1)$
Schwefel	[−500, 500]	$f_{8}(\vec {x})=\sum _{i=1}^{D}\left ({-x_{i}sin\left ({\sqrt {\left |{ x_{i} }\right |} }\right) }\right)$
Rastrigin	[−5.12, 5.12]	$f_{9}(\vec {x})=\sum _{i=1}^{D}\left ({x_{i}^{2}-10cos\left ({2\pi x_{i} }\right) +10 }\right)$
Ackley	[−32, 32]	$f_{10}(\vec {x})= 20 + e - 20exp\left ({-0.2\sqrt {\frac {1}{D}\sum _{i=1}^{D}x_{i}^{2}} }\right) - exp\left ({\frac {1}{D}\sum _{i=1}^{D}cos\left ({2\pi x_{i} }\right) }\right)$
Griewank	[−600, 600]	$f_{11}(\vec {x})=\frac {1}{4000}\left ({\sum _{i=1}^{D}x_{i}^{2},}\right)-\left ({\prod _{i=1}^{D}cos\left ({\frac {x_{i}}{\sqrt {i}} }\right) }\right)+1 $
Penalized	[−50, 50]	$\begin{aligned} f_{12}(\vec{x})=\frac{\pi}{D}\left(10 \sin ^{2}\left(\pi y_{i}\right)+\left(\sum_{i=1}^{D}\left(y_{i}-1\right)^{2}\left(1+10 \sin ^{2}\left(\pi y_{i+1}\right)\right)\right)\right. \\ \sum_{i=1}^{D} u\left(x_{i}, 10,100,4\right) \\ u\left(x_{i}, a, k, m\right)=\left\{\begin{array}{cc} k\left(x_{i}-a\right)^{m}, & x_{i}\gt a \\ 0, & -a \leq x_{i} \leq a \\ k\left(-x_{i}-a\right)^{m}, & x_{i} \end{array}\right\} \quad y_{i}=1+\frac{1}{4}\left(x_{i}+1\right) \end{aligned}$
Penalized1	[−50, 50]	$\begin{aligned} f_{13}(\vec{x})=0.1\left(10 \sin ^{2}\left(\pi y_{i}\right)+\left(\sum_{i=1}^{D}\left(y_{i}-1\right)^{2}\left(1+10 \sin ^{2}\left(\pi y_{i+1}\right)\right)\right)\right. \\ \sum_{i=1}^{D} u\left(x_{i}, 10,100,4\right) \\ u\left(x_{i}, a, k, m\right)=\left\{\begin{array}{cc} k\left(x_{i}-a\right)^{m}, & x_{i}\gt a \\ 0, & -a \leq x_{i} \leq a \\ k\left(-x_{i}-a\right)^{m}, & x_{i} \end{array}\right\} \quad y_{i}=1+\frac{1}{4}\left(x_{i}+1\right) \end{aligned}$

**TABLE 2:** Results of the IP Algorithm With Different $NoR$ and $NoD$ Parameter Values

Fn.		$NoR=1$			$NoR=2$			$NoR=3$		
		$NoD=1$	$NoD=2$	$NoD=3$	$NoD=1$	$NoD=2$	$NoD=3$	$NoD=1$	NoD=2	$NoD=3$
$f_{1}$	Mean	4.584737e−270	3.789457e−264	7.622723e−210	1.477167e−247	0.000000e+00	0.000000e+00	1.596746e−223	0.000000e+00	0.000000e+00
	Std.	0.000000e+00	0.000000e+00	0.000000e+00	0.000000e+00	0.000000e+00	0.000000e+00	0.000000e+00	0.000000e+00	0.000000e+00
$f_{2}$	Mean	6.984169e−231	2.567990e−196	5.248317e−155	2.850815e−227	2.769813e−282	2.755805e−250	2.075237e−219	3.463669e−289	3.792181e−302
	Std.	0.000000e+00	0.000000e+00	2.823289e−154	0.000000e+00	0.000000e+00	0.000000e+00	0.000000e+00	0.000000e+00	0.000000e+00
$f_{3}$	Mean	1.774811e−143	0.000000e+00	0.000000e+00	2.394175e+02	1.936706e−283	0.000000e+00	3.236258e+02	2.734727e+00	0.000000e+00
	Std.	9.637998e−143	0.000000e+00	0.000000e+00	1.037086e+03	0.000000e+00	0.000000e+00	1.195234e+03	8.206728e+00	0.000000e+00
$f_{4}$	Mean	2.008090e−47	1.233475e−153	1.462673e−168	7.173733e−72	1.569312e−186	7.237433e−260	8.235588e−30	9.620043e−171	5.683667e−262
	Std.	1.099876e−46	6.756023e−153	0.000000e+00	2.773148e−71	0.000000e+00	0.000000e+00	4.510792e−29	0.000000e+00	0.000000e+00
$f_{5}$	Mean	5.477595e−02	2.799752e+01	2.826561e+01	3.598711e+01	1.048155e+00	2.779750e+01	5.277339e+01	3.486574e+01	1.139031e+01
	Std.	1.086144e−01	8.637723e−02	1.651070e−01	6.043880e+01	2.263503e+00	1.259441e−01	3.283248e+01	2.905658e+01	1.765911e+01
$f_{6}$	Mean	0.000000e+00	0.000000e+00	0.000000e+00	0.000000e+00	0.000000e+00	0.000000e+00	0.000000e+00	0.000000e+00	0.000000e+00
	Std.	0.000000e+00	0.000000e+00	0.000000e+00	0.000000e+00	0.000000e+00	0.000000e+00	0.000000e+00	0.000000e+00	0.000000e+00
$f_{7}$	Mean	2.154104e−04	1.033819e−05	1.256575e−05	9.948510e−04	1.823647e−04	1.417827e−06	1.894527e−03	7.819080e−04	2.975616e−04
	Std.	3.430556e−04	1.818616e−05	2.053878e−05	8.998333e−04	2.383373e−04	5.669494e−06	2.970506e−03	1.042715e−03	5.500870e−04
$f_{8}$	Mean	-1.256554e+04	-5.779741e+03	-5.737285e+03	-1.046854e+04	-1.251024e+04	-5.833471e+03	-1.055187e+04	-1.077194e+04	-1.224037e+04
	Std.	2.162409e+01	2.237178e+02	1.825124e+02	3.669849e+02	1.066222e+02	1.959748e+02	4.533353e+02	2.715433e+02	3.448724e+02
$f_{9}$	Mean	0.000000e+00	0.000000e+00	0.000000e+00	0.000000e+00	0.000000e+00	0.000000e+00	0.000000e+00	0.000000e+00	0.000000e+00
	Std.	0.000000e+00	0.000000e+00	0.000000e+00	0.000000e+00	0.000000e+00	0.000000e+00	0.000000e+00	0.000000e+00	0.000000e+00
$f_{10}$	Mean	4.440892e−16	4.440892e−16	4.440892e−16	4.440892e−16	4.440892e−16	4.440892e−16	4.440892e−16	4.440892e−16	4.440892e−16
	Std.	1.002933e−31	1.002933e−31	1.002933e−31	1.002933e−31	1.002933e−31	1.002933e−31	1.002933e−31	1.002933e−31	1.002933e−31
$f_{11}$	Mean	0.000000e+00	0.000000e+00	0.000000e+00	0.000000e+00	0.000000e+00	0.000000e+00	0.000000e+00	0.000000e+00	0.000000e+00
	Std.	0.000000e+00	0.000000e+00	0.000000e+00	0.000000e+00	0.000000e+00	0.000000e+00	0.000000e+00	0.000000e+00	0.000000e+00
$f_{12}$	Mean	1.578347e−32	9.730136e−02	4.575892e−01	6.960183e−03	1.695838e−30	1.342649e−01	4.440423e−03	3.745442e−06	7.882587e−14
	Std.	1.545535e−34	3.638779e−02	8.436380e−02	2.629163e−02	2.985404e−30	2.987308e−02	2.128388e−02	1.150514e−05	4.317471e−13
$f_{13}$	Mean	1.502115e−32	8.932541e−02	4.122044e−01	3.304023e−03	2.090997e−30	1.161165e−01	1.041948e−05	3.701284e−04	2.509591e−07
	Std.	4.281179e−35	3.767177e−02	6.021590e−02	1.807447e−02	3.978424e−30	3.382516e−02	2.212770e−05	1.409899e−03	1.374245e−06

The results given in the Table 2 give important information about the appropriate $NoR$ and $NoD$ combination of the IP algorithm. While the IP algorithm is capable of obtaining global best solutions of the $f_{6}$, $f_{9}$, $f_{10}$ and $f_{11}$ functions for all of the nine $NoR$ and $NoD$ combinations, more qualified solutions of the $f_{1}$, $f_{2}$, $f_{3}$, $f_{4}$, $f_{5}$, $f_{7}$, $f_{8}$, $f_{12}$ and $f_{13}$ functions can be found by the IP algorithm with the subtly determined $NoR$ and $NoD$ parameters. For $f_{5}$, $f_{8}$, $f_{12}$ and $f_{13}$ functions, IP algorithm shows better performance by setting both $NoR$ and $NoD$ parameters to their smallest value compared to the other parameter configurations. For $f_{1}$ function, if there is more than one receiver in other words the $NoR$ is bigger than 1, the $NoD$ parameter should be chosen equal or bigger than the $NoR$ parameter. Similar generalizations can also be made about the $f_{3}$ function by considering the obtained results. For this benchmark function, the IP algorithm should set the $NoD$ parameter higher than the $NoR$ parameter. Finally, for the $f_{2}$ and $f_{4}$ functions, the IP algorithm produce better results by setting the $NoR$ and $NoD$ to 2 or 3 when compared to the other $NoR$ and $NoD$ configurations.

**FIGURE 4.**
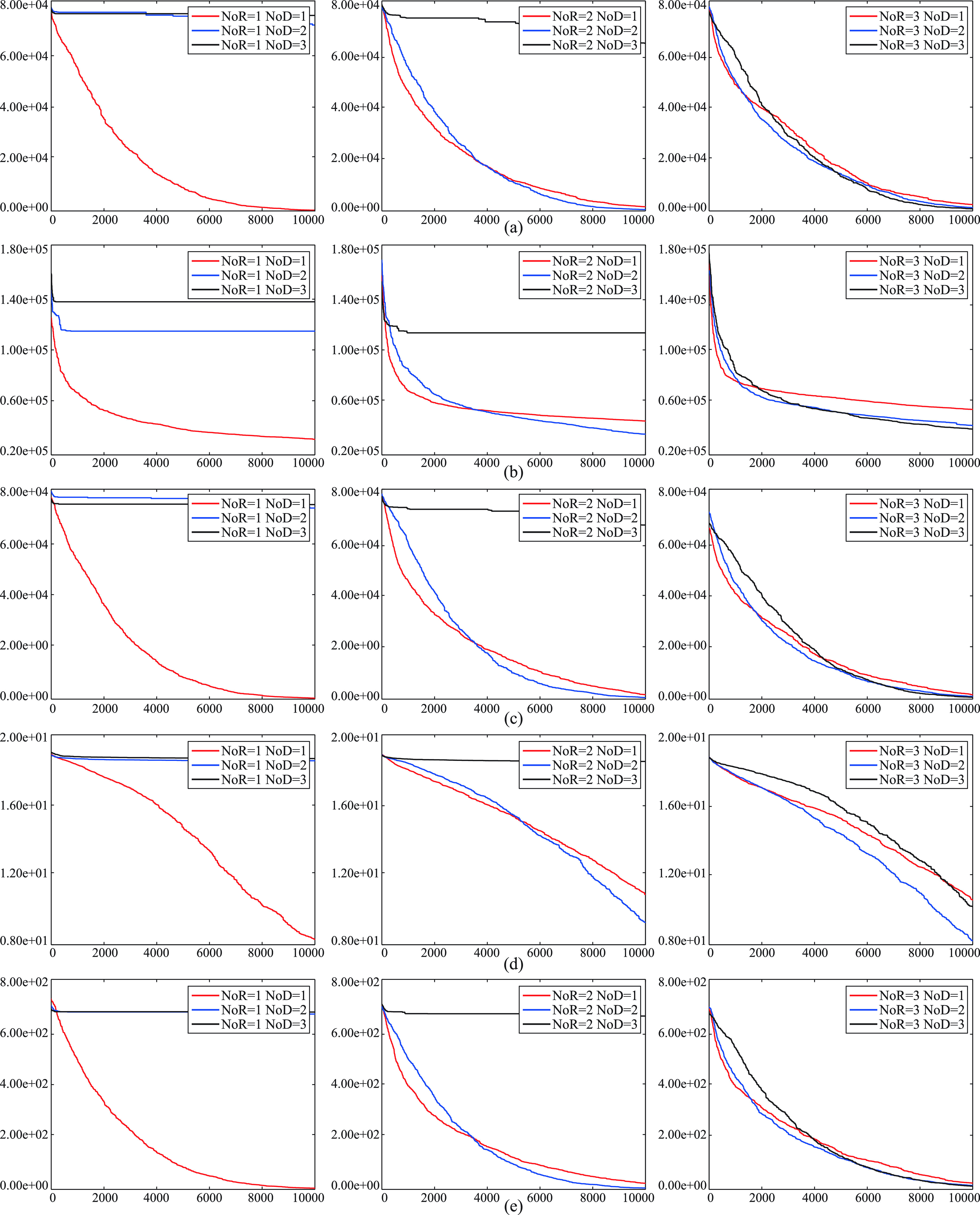
Convergence curves of the IP algorithm with varying $NoD$ parameters for the 30-dimensional $f_{1}$ (a), $f_{3}$ (b), $f_{6}$ (c), $f_{10}$ (d) and $f_{11}$ (e) functions.

**FIGURE 5.**
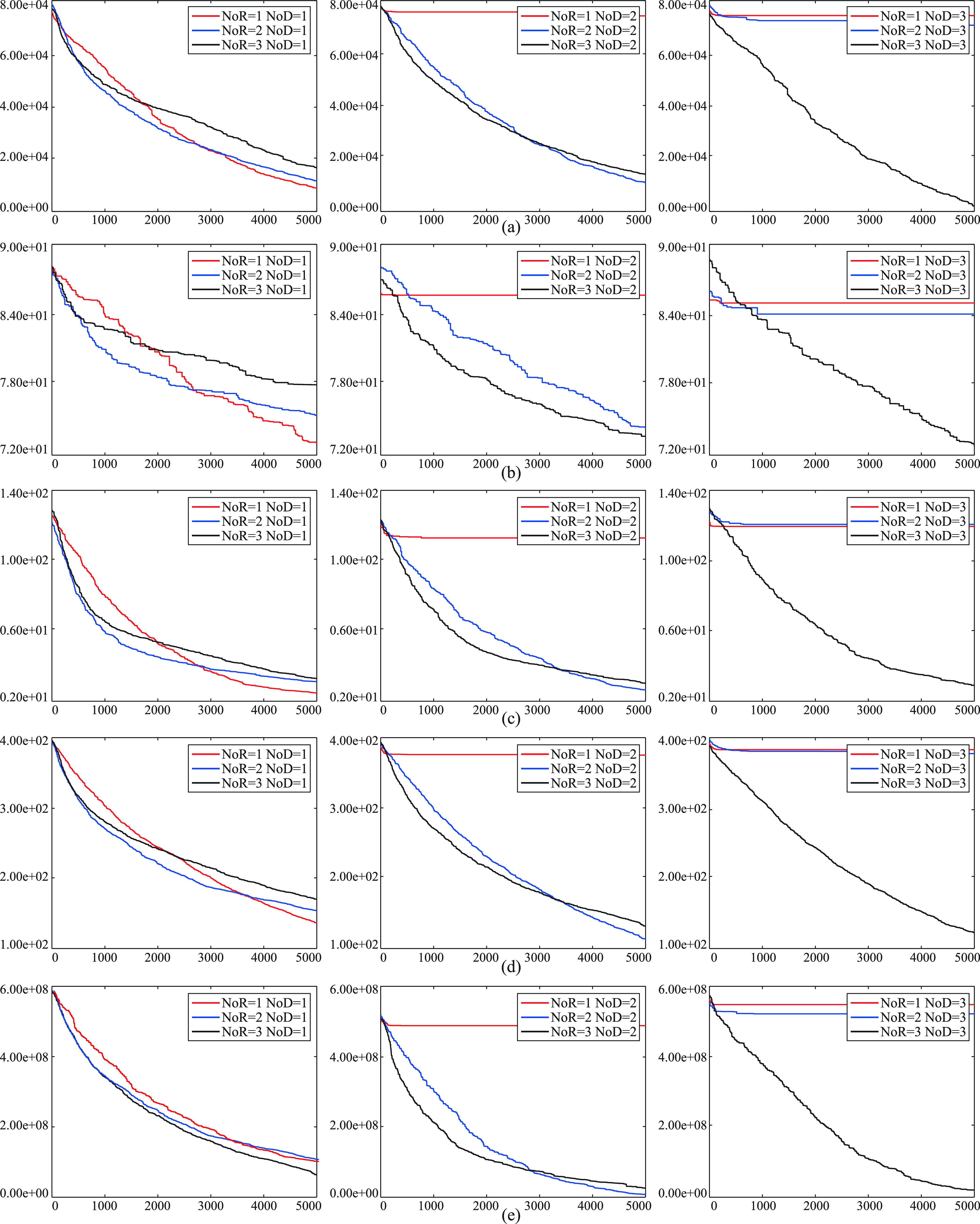
Convergence curves of the IP algorithm with varying $NoR$ parameters for the 30-dimensional $f_{1}$ (a), $f_{4}$ (b), $f_{7}$ (c), $f_{9}$ (d) and $f_{12}$ (e) functions.

The values assigned to the $NoR$ and $NoD$ parameters of the IP algorithm have an impact on the convergence performance of it. For providing a visual representation about the convergence characteristics of the IP algorithm with varying $NoR$ and $NoD$ parameters, some curves are plotted and illustrated in the Fig. 4 and Fig. 5. In the Fig. 4, the $NoR$ parameter is set to a fixed value while the $NoD$ parameter is changed. In the Fig. 5, the $NoR$ parameter is changed while the $NoD$ parameter is set to a fixed value. The curves of the figures help to make a rough generalization about that the IP algorithm converges more quickly when the $NoD$ parameter is chosen equal or less than the $NoR$ parameter. As mentioned before, the IP algorithm supports the a receiver with the plasma of the selected donor. If most of the receivers are supported with the plasma taken from the same donor, the neighborhood of the solution represented with the selected donor is examined continuously and the convergence speed of the IP algorithm is accelerate intrinsically. Even though there are more than one donor with the condition that number of donors is equal or less than the number of receivers, examining donors with the similar qualities still contributes to the performance of the IP algorithm. However, it should be noticed that the convergence speed accelerated at the initial cycles with the usage of small $NoD$ parameter can cause trapping local optimums especially for the problems in which there are local optimums relatively close to the global optimum or optimums.

In order to analyze how the qualities of the final solutions of the IP algorithm change with the increased population sizes, two different values including 50 and 100 are assigned to the $PS$ and the benchmark functions given in the Table 1 are solved again. The total evaluation number is set to 150,000 for the $f_{1}$, $f_{6}$, $f_{10}$, $f_{12}$ and $f_{13}$ functions, 200,000 for the $f_{2}$ and $f_{11}$ functions, 300,000 for the $f_{7}$, $f_{8}$ and $f_{9}$ functions. Finally, the total evaluation number is set to 500,000 for the $f_{3}$, $f_{4}$ and $f_{5}$ functions. The $NoR$ parameter is set to 1 and the $NoD$ parameter is set to 1 and 2, respectively. For each benchmark function, 30 independent runs with random seeds are carried out. The mean best objective function values and standard deviations are recorded and summarized in the Table 3. When the results given in the Table 3 are investigated, it is clearly seen that the IP algorithm with the increasing population size still protects its performance on the $f_{6}$, $f_{9}$, $f_{10}$ and $f_{11}$ functions. While the IP algorithm finds more qualified solutions for the $f_{5}$ and $f_{8}$ functions with the $PS$ equal to 50, the population size equal to 30 is found more appropriate for the $f_{1}$, $f_{2}$, $f_{3}$, $f_{4}$, $f_{7}$, $f_{12}$ and $f_{13}$ functions. Because of the different characteristics of the used benchmark functions, the requirements of the IP algorithm to do with the $PS$, $NoR$ and $NoD$ parameters can be vary. However, the results given in the Table 2 and Table 3 help for making a generalization that the $PS$ parameter can be chosen between between 30 and 50 and the $NoR$ and $NoD$ parameters can be set to 1 or 2.

**TABLE 3:** Results of the IP Algorithm With Different $PS$, $NoR$ and $NoD$ Parameter Values

Fn.		$PS=30$		$PS=50$		$PS=100$	
		$NoR=1$ and $NoD=1$	$NoR=1$ and $NoD=2$	$NoR=1$ and $NoD=1$	$NoR=1$ and $NoD=2$	$NoR=1$ and $NoD=1$	$NoR=1$ and $NoD=2$
$f_{1}$	Mean	4.584737e−270	3.789457e−264	2.034721e−156	4.278673e−157	8.920118e−70	9.683726e−77
	Std.	0.000000e+00	0.000000e+00	7.743299e−156	2.067361e−156	3.845662e−69	5.301682e−76
$f_{2}$	Mean	6.984169e−231	2.567990e−196	9.680051e−139	1.847288e−121	2.293902e−66	6.057797e−58
	Std.	0.000000e+00	0.000000e+00	5.295174e−138	9.819635e−121	1.178645e−65	3.193784e−57
$f_{3}$	Mean	1.774811e−143	0.000000e+00	7.797258e−66	4.518427e−275	2.962588e−26	2.627849e−122
	Std.	9.637998e−143	0.000000e+00	4.270554e−65	0.000000e+00	1.594136e−25	1.439332e−121
$f_{4}$	Mean	2.008090e−47	1.233475e−153	1.348303e−16	2.372519e−75	1.223419e−02	4.547725e−27
	Std.	1.099876e−46	6.756023e−153	5.334654e−16	1.299482e−74	2.496071e−02	1.743169e−26
$f_{5}$	Mean	5.477595e−02	2.799752e+01	2.499217e−02	2.782835e+01	3.570249e+00	2.779294e+01
	Std.	1.086144e−01	8.637723e−02	5.059487e−02	3.516931e−01	8.465702e+00	2.340188e−01
$f_{6}$	Mean	0.000000e+00	0.000000e+00	0.000000e+00	0.000000e+00	0.000000e+00	0.000000e+00
	Std.	0.000000e+00	0.000000e+00	0.000000e+00	0.000000e+00	0.000000e+00	0.000000e+00
$f_{7}$	Mean	2.154104e−04	1.033819e−05	1.958954e−04	7.491989e−05	3.204142e−04	1.823603e−04
	Std.	3.430556e−04	1.818616e−05	1.976426e−04	9.536009e−05	3.832165e−04	1.623893e−04
$f_{8}$	Mean	-1.256554e+04	-5.779741e+03	-1.256949e+04	-5.665578e+03	-1.244280e+04	-6.186269e+03
	Std.	2.162409e+01	2.237178e+02	5.550256e−12	1.479207e+02	8.560684e+01	1.136933e+02
$f_{9}$	Mean	0.000000e+00	0.000000e+00	0.000000e+00	0.000000e+00	0.000000e+00	0.000000e+00
	Std.	0.000000e+00	0.000000e+00	0.000000e+00	0.000000e+00	0.000000e+00	0.000000e+00
$f_{10}$	Mean	4.440892e−16	4.440892e−16	4.440892e−16	4.440892e−16	7.993606e−16	4.440892e−16
	Std.	1.002933e−31	1.002933e−31	1.002933e−31	1.002933e−31	1.084035e−15	1.002933e−31
$f_{11}$	Mean	0.000000e+00	0.000000e+00	0.000000e+00	0.000000e+00	0.000000e+00	0.000000e+00
	Std.	0.000000e+00	0.000000e+00	0.000000e+00	0.000000e+00	0.000000e+00	0.000000e+00
$f_{12}$	Mean	1.578347e−32	9.730136e−02	6.862657e−21	1.950335e−02	9.484211e−10	9.601333e−03
	Std.	1.545535e−34	3.638779e−02	7.066457e−21	1.027764e−02	5.565435e−10	6.919741e−03
$f_{13}$	Mean	1.502115e−32	8.932541e−02	4.934411e−21	1.891750e−02	1.079312e−09	8.044402e−03
	Std.	4.281179e−35	3.767177e−02	6.812016e−21	1.023187e−02	7.973110e−10	4.540242e−03

For verifying the results of the IP algorithm, it is compared with the PSO [11], DE [7], RCBBO [9], CS [12], FA [13], GSA [34], ABC [16] and AMO [50] algorithms. The population or colony size of these algorithms is taken equal to 50 or 100 for setting the number of evaluations to 100 per cycle or iteration [50]. In the comparisons, the population size of the IP algorithm is also taken equal to 50. The $NoR$ parameter is set to 1 for each benchmark function. Moreover, the $NoD$ parameter is set to 2 for $f_{3}$, $f_{4}$ and $f_{7}$ functions while it is set to 1 for the other benchmark functions. The total evaluation number is set to 150,000 for the $f_{1}$, $f_{6}$, $f_{10}$, $f_{12}$ and $f_{13}$ functions, 200,000 for the $f_{2}$ and $f_{11}$ functions, 300,000 for the $f_{7}$, $f_{8}$ and $f_{9}$ functions, 500,000 for the $f_{3}$, $f_{4}$ and $f_{5}$ functions [50]. The mean best objective function values of the 30 independent runs and standard deviations are summarized in the Table 4.

**TABLE 4:** Comparison Between IP Algorithm and Other Meta-Heuristics for 30 Dimensional Problems

Fn.		IPA	PSO [11], [50]	DE [7], [50]	RCBBO [9], [50]	CS [12], [50]	FA [13], [50]	GSA [34], [50]	ABC [16], [50]	AMO [50]
$f_{1}$	Mean	2.0347e−156	3.3340e−10	5.6016e−14	3.7370e−01	5.6565e−06	1.7000e−03	3.3748e−18	2.9860e−20	8.6464e−40
	Std.	7.7432e−156	7.0387e−10	4.4053e−14	1.1810e−01	2.8611e−06	4.0608e−04	8.0862e−19	2.1455e−20	1.0435e−39
	Rank	1	6	5	9	7	8	4	3	2
$f_{2}$	Mean	9.6800e−139	6.6598e−11	4.7348e−10	1.6560e−01	2.0000e−03	4.5300e−02	8.9211e−09	1.4213e−15	8.2334e−32
	Std.	5.2951e−138	9.2553e−11	1.7759e−10	3.4200e−02	8.0959e−04	3.3800e−02	1.3340e−09	5.5340e−16	3.4120e−32
	Rank	1	4	5	9	7	8	6	3	2
$f_{3}$	Mean	4.5184e−275	2.9847e+00	2.8038e−11	1.5972e+03	1.4000e−03	1.8200e−02	1.1260e−01	2.4027e+03	8.8904e−04
	Std.	0.0000e+00	2.2778 e+00	3.6788e−11	8.3360e+02	6.0987e−04	6.4000e−03	1.2660e−01	6.5696e+02	8.7256e−04
	Rank	1	7	2	8	4	5	6	9	3
$f_{4}$	Mean	2.3725e−75	7.9997e+00	2.2160e−01	7.9738e+00	3.2388e+00	5.5400e−02	9.9302e−10	1.8522e+01	2.8622e−05
	Std.	1.2994e−74	2.5351e+00	2.4300e−01	2.6633e+00	6.6440e−01	1.0100e−02	1.1899e−10	4.2477e+00	2.3468e−05
	Rank	1	8	5	7	6	4	2	9	3
$f_{5}$	Mean	2.4992e−02	4.69202e+01	2.6570e−01	6.4690e+01	8.0092e+00	3.81248e+01	2.0081e+01	4.4100e−02	4.1817e+00
	Std.	5.0594e−02	3.80312 e+01	1.0293e+00	3.6278e+01	1.9188e+00	3.03962e+01	1.7220e−01	7.0700e−02	2.1618e+00
	Rank	1	5	3	9	6	8	7	2	4
$f_{6}$	Mean	0.0000e+00	3.6925e−10	4.5028e−14	3.6950e−01	5.4332e−06	1.7000e−03	3.3385e−18	3.0884e−20	0.0000e+00
	Std.	0.0000e+00	6.3668e−10	2.3309e−14	1.1150e−01	2.2446e−06	4.1593e−04	5.6830e−19	4.0131e−20	0.0000e+00
	Rank	1	6	5	9	7	8	4	3	1
$f_{7}$	Mean	7.4919e−05	1.3500e−02	4.2000e−03	3.0000e−03	9.6000e−03	8.2000e−03	3.9000e−03	3.2400e−02	1.7000e−03
	Std.	9.5360e−05	4.1000e−03	1.4000e−03	1.2000e−03	2.8000e−03	9.3000e−03	1.3000e−03	5.9000e−03	4.7058e−04
	Rank	1	8	5	3	7	6	4	9	2
$f_{8}$	Mean	-1.2569e+04	-8.8278e+03	-1.1276e+04	-1.2568e+04	-9.1492e+03	-6.2238e+03	-3.0499e+03	-1.2507e+04	-1.2569e+04
	Std.	5.5502e−12	6.1115e+02	1.8135e+03	5.7580e−01	2.5314e+02	7.7230e+02	3.3886e+02	6.1118e+01	1.2384e−07
	Rank	1	7	5	3	6	8	9	4	1
$f_{9}$	Mean	0.0000e+00	1.8267e+01	1.3467e+02	3.8500e−02	5.1220e+01	2.35213e+01	7.2831e+00	0.0000e+00	0.0000e+00
	Std.	0.0000e+00	4.796e+00	2.8859e+01	1.5400e−02	8.1069e+00	8.3683e+00	1.8991e+00	0.0000e+00	0.0000e+00
	Rank	1	6	9	4	8	7	5	1	1
$f_{10}$	Mean	4.4408e−16	3.8719e−06	7.4739e−08	1.9470e−01	2.3750e+00	9.4000e−03	1.4717e−09	1.1946e−09	4.4409e−15
	Std.	1.0029e−31	2.8604e−06	3.1082e−08	4.6100e−02	1.1238e+00	1.4000e−03	1.4449e−10	5.0065e−10	0.0000e+00
	Rank	1	6	5	8	9	7	4	3	2
$f_{11}$	Mean	0.0000e+00	1.6800e−02	0.0000e+00	2.7650e−01	4.4900e−05	2.5000e−03	1.2650e−02	0.0000e+00	0.0000e+00
	Std.	0.0000e+00	2.0500e−02	0.0000e+00	7.9600e−02	8.9551e−05	4.6910e−04	2.1600e−02	0.0000e+00	0.0000e+00
	Rank	1	8	1	9	5	6	7	1	1
$f_{12}$	Mean	6.8626e−21	8.3000e−03	4.7114e−15	2.0000e−03	5.0710e−01	8.8694e−06	2.0358e−20	1.1928e−21	1.5705e−32
	Std.	7.0664e−21	2.8700e−02	3.2597e−15	2.3000e−03	2.6620e−01	2.7999e−06	4.5322e−21	1.0783e−21	2.8080e−48
	Rank	3	8	5	7	9	6	4	2	1
$f_{13}$	Mean	4.9344e−21	4.6694e−07	3.1598e−14	2.1800e−02	4.6965e−04	1.2812e−04	5.6991e−33	2.2990e−20	1.3498e−32
	Std.	6.8120e−21	1.3713e−06	2.2825e−14	9.6000e−03	2.9932e−04	4.1539e−05	6.2589e−33	2.2886e−20	2.8080e−48
	Rank	3	6	5	9	8	7	1	4	2
Average Rank		1.3077	6.5385	4.6154	7.2308	6.8462	6.7692	4.8462	4.0769	1.9231
Overall Rank		1	6	4	9	8	7	5	3	2

From the results given in the Table 4, it is seen that the IP algorithm produces equal or better solutions than the compared meta-heuristics for eleven of the thirteen benchmark functions and shows its comparative performance with the average rank found equal to 1.3077 and overall rank found equal to 1. Only for the $f_{12}$ and $f_{13}$ functions, the IP algorithm lags behind two meta-heuristics and its rank is determined as 3. The idea lying behind the distribution of the infection between individuals provides an efficient exploration capability for the IP algorithm. In addition to this, the plasma transfer mechanism that starts with the determination of the receiver and donor individuals, continues with the treatment of the receiver or receivers and terminates with adjustment of antibody density of the donor or donors significantly contributes to the exploitation characteristic of the IP algorithm. The effect of this unique mechanism of the IP algorithm becomes more apparent for the $f_{1}$, $f_{2}$, $f_{3}$, $f_{4}$, $f_{7}$ and $f_{10}$ benchmark functions.

The mean best objective function values, average and overall ranks of the IP algorithm show its comparative performance. However, some statistical evidences about the performance of the IP algorithm should be provided. In order to decide that the solutions found by the IP algorithm are strongly enough to prove the efficiency of the IP algorithm with statistical outputs, the Wilcoxon signed-rank test that is one of the most commonly used non-parametric tests is used. In the Wilcoxon signed-rank test, the significance level showed by $\rho $ is taken equal to 0.05. If the $\rho $ value calculated for the two competitors is less than 0.05, it is accepted that there is a statistical difference between competitors in favor of one of them. Otherwise, it is said that the statistical difference between two competitors is not meaningful for deciding in favor of one of them. The Wilcoxon signed-rank test results between IP algorithm and other tested meta-heuristics are given in the Table (5). In Table (5), $W+$ column corresponds to the sum of the ranks for which IP algorithm is worse than the compared meta-heuristic. Similarly, $W-$ column shows the sum of the ranks for which IP algorithm is better than the compared meta-heuristic. Finally, $Z$-value is equal to the mean difference between IP algorithm and other technique. When the test results summarized in the Table (5) are investigated, it is seen that the superiority of the IP algorithm is also proven statistically. While the statistical significance is in favor of IP algorithm compared to the PSO, DE, RCBBO, CS, FA, GSA and ABC, there is no statistical difference between IP and AMO algorithms. Even though the results found by the IP algorithm are better or equal to the results of the AMO for eleven of thirteen problems, they are not enough to generate a statistically significant difference.

**TABLE 5:** Statistical Comparison Between Meta-Heuristics for 30 Dimensional Problems

IPA vs.	Sum of Ranks		$Z$-val.	$\rho $-val.	Sign.
	$W+$	$W-$			
PSO	12	66	2.07963	0.01877	IPA
DE	11	55	1.91252	0.02790	IPA
RCBBO	12	66	2.07963	0.01877	IPA
CS	12	66	2.07963	0.01877	IPA
FA	12	66	2.08043	0.01874	IPA
GSA	12	66	2.07963	0.01877	IPA
ABC	10	45	1.73392	0.04146	IPA
AMO	18	47	1.24598	0.10638	–

Another investigation about the IP algorithm is carried out how the execution time of it changes with the different values assigned to the $PS$, $NoR$ and $NoD$ parameters. For each 30-dimensional benchmark problem, the IP algorithm coded with C programming language is tested 30 different times on a PC equipped with a single core 1.33 GHz Intel processor by using the previously mentioned termination criteria. The average execution times in terms of second over the 30 independent runs and related standard deviations are calculated and summarized at the Table 6. The results given in the Table 6 show that the execution time of the IP algorithm increases slightly with the size of population even though the termination criteria is same. The main reason lying behind this kind of change is directly related with the donor and receiver selection. When the population size is increased from 30 to 50 or 100, determination of the donor and receiver individuals requires more comparisons and brings extra computational burden to the execution time of the IP algorithm. Another important situation that should be considered when analyzing the execution time of the IP algorithm is the number of donor and receiver individuals. The results in the Table 6 indicate that the number of donor and receiver individuals does not increase the execution time of the IP algorithm substantially compared to the selection of the donor and receiver individuals.

**TABLE 6:** Average Execution Times of the IP Algorithm for 30 Dimensional Problems

Fn.		$PS=30$		$PS=50$		$PS=100$	
		$NoR=1$		$NoR=1$		$NoR=1$	
		$NoD=1$	$NoD=2$	$NoD=1$	$NoD=2$	$NoD=1$	$NoD=2$
$f_{1}$	Mean	0.231	0.259	0.257	0.288	0.340	0.334
	Std.	6.227e-02	2.403e-02	2.498e-02	3.063e-02	4.398e-02	3.748e-02
$f_{2}$	Mean	0.391	0.390	0.398	0.414	0.476	0.440
	Std.	3.772e-02	7.006e-02	2.203e-02	3.481e-02	2.471e-02	3.623e-02
$f_{3}$	Mean	4.368	4.424	4.461	4.727	4.790	4.843
	Std.	2.839e-01	2.087e-01	4.661e-01	5.499e-01	2.802e-01	2.586e-01
$f_{4}$	Mean	0.752	0.766	0.954	0.877	1.056	0.935
	Std.	2.218e-02	4.679e-02	1.688e-01	2.995e-02	4.508e-02	7.168e-02
$f_{5}$	Mean	1.055	1.122	1.380	1.176	1.414	1.435
	Std.	2.848e-02	3.933e-02	4.900e-02	4.738e-02	4.922e-02	1.633e-01
$f_{6}$	Mean	0.963	0.982	1.103	1.022	1.146	1.118
	Std.	2.021e-02	2.720e-02	3.561e-02	9.500e-02	1.103e-01	1.817e-01
$f_{7}$	Mean	1.751	1.895	2.070	1.905	2.122	1.982
	Std.	6.892e-02	3.456e-01	1.049e-01	2.288e-01	2.786e-01	2.650e-01
$f_{8}$	Mean	1.906	1.843	1.992	1.867	2.085	1.932
	Std.	2.359e-01	7.934e-02	2.223e-01	3.898e-02	4.353e-01	1.576e-01
$f_{9}$	Mean	1.731	1.783	1.914	1.922	2.073	1.967
	Std.	9.404e-02	2.420e-01	3.497e-01	2.851e-01	4.132e-01	4.425e-02
$f_{10}$	Mean	1.430	1.397	1.465	1.485	1.526	1.580
	Std.	1.733e-01	2.200e-01	1.351e-01	1.103e-01	5.229e-02	1.246e-01
$f_{11}$	Mean	2.776	2.642	3.257	3.250	3.398	3.523
	Std.	1.381e-01	2.064e-01	6.062e-01	6.984e-01	5.392e-01	3.442e-01
$f_{12}$	Mean	2.379	2.501	2.458	2.633	2.754	2.737
	Std.	7.689e-02	1.794e-01	1.865e-01	3.692e-01	1.944e-01	1.282e-01
$f_{13}$	Mean	2.379	2.575	2.497	2.599	2.554	2.675
	Std.	8.987e-02	3.246e-02	2.977e-01	8.347e-02	1.722e-01	1.189e-01

### B. Solving High-Dimensional Benchmark Problems With IP Algorithm

The results of the IP algorithm for 30 dimensional benchmark functions provide information about the comparative and promising performance of it. However, the performance of the IP algorithm should also be evaluated on solving high dimensional problems and comparative studies should be carried out for deciding whether the mechanisms of the IP algorithm are still useful or not. For this purpose, the benchmark functions ranging from $f_{1}$ to $f_{12}$ given in the Table 1 are solved with the IP algorithm by setting the number of parameters of to 100 and compared with the MFO [18], PSO [11], GSA [34], BA [14], FPA [15], SMS [38], FA [13] and GA [6]. In order to provide a fair comparison with the MFO, PSO, GSA, BA, FPA, SMS, FA and GA, the population size of the IP is 30 and total evaluation number is 30,000 for each benchmark function [18]. The $NoR$ parameter of the IP algorithm is taken equal to 1 for each benchmark function. Moreover, the $NoD$ parameter is set to 2 for $f_{3}$, $f_{4}$ and $f_{9}$ functions while it is set to 1 for the other benchmark functions. When the results calculated after 30 different runs in the Table 7 is analyzed, it is understood that the IP algorithm still protects its solving capabilities even though the dimensionality of the problems is increased. For ten of the twelve benchmark functions, the IP algorithm outperforms other competitors and its overall rank is determined as 1. While the IP algorithm is the third best method for the $f_{4}$ on the basis of the mean objective function values, it lags behind GSA, MFO, FPA and PSO for $f_{12}$ function and its rank is determined as 5.

**TABLE 7:** Comparison Between IP Algorithm and Other Meta-Heuristics for 100 Dimensional Problems

Fn.		IPA	MFO [18]	PSO [11], [18]	GSA [18], [34]	BA [14], [18]	FPA [15], [18]	SMS [18], [38]	FA [13], [18]	GA [6], [18]
$f_{1}$	Mean	7.4671e−27	1.1700e−04	1.3212e+00	6.0823e+02	2.0792e+04	2.0364e+02	1.2000e+02	7.4807e+03	2.1886e+04
	Std.	1.9330e−26	1.5000e−04	1.1539e+00	4.6465e+02	5.8924e+03	7.8398e+01	0.0000e+00	8.9485e+02	2.8796e+03
	Rank	1	2	3	6	8	5	4	7	9
$f_{2}$	Mean	9.2362e−26	6.3900e−04	7.7156e+00	2.2753e+01	8.9786e+01	1.1169e+01	2.0531e−02	3.9325e+01	5.6518e+01
	Std.	2.2260e−25	8.7700e−04	4.1321e+00	3.3651e+00	4.1958e+01	2.9196e+00	4.7180e−03	2.4659e+00	5.6609e+00
	Rank	1	2	4	6	9	5	3	7	8
$f_{3}$	Mean	4.5045e−14	6.9673e+02	7.3639e+02	1.3576e+05	6.2481e+04	2.3757e+02	3.7820e+04	1.7357e+04	3.7010e+04
	Std.	2.4546e−13	1.8853e+02	3.6178e+02	4.8653e+04	2.9769e+04	1.3665e+02	0.0000e+00	1.7401e+03	5.5722e+03
	Rank	1	3	4	9	8	2	7	5	6
$f_{4}$	Mean	2.3461e+01	7.0686e+01	1.2973e+01	7.8782e+01	4.9743e+01	1.2573e+01	6.9170e+01	3.3954e+01	5.9143e+01
	Std.	1.6448e+01	5.2751e+00	2.6344e+00	2.8141e+00	1.0144e+01	2.2900e+00	3.8767e+00	1.8697e+00	4.6485e+00
	Rank	3	8	2	9	5	1	7	4	6
$f_{5}$	Mean	1.1020e+02	1.3915e+02	7.7361e+04	7.4100e+02	1.9951e+06	1.0975e+04	6.3822e+06	3.7950e+06	3.1321e+07
	Std.	4.3071e+01	1.2026e+02	5.1156e+04	7.8124e+02	1.2524e+06	1.2057e+04	7.2997e+05	7.5903e+05	5.2645e+06
	Rank	1	2	5	3	6	4	8	7	9
$f_{6}$	Mean	0.0000e+00	1.1300e−04	2.8665e+02	3.0810e+03	1.7053e+04	1.7538e+02	4.1439e+04	7.8287e+03	2.0965e+04
	Std.	0.0000e+00	9.8700e−05	1.0708e+02	8.9863e+02	4.9176e+03	6.3453e+01	3.2952e+03	9.7521e+02	3.8681e+03
	Rank	1	2	4	5	7	3	9	6	8
$f_{7}$	Mean	7.0755e−03	9.1155e−02	1.0373e+00	1.1298e−01	6.0451e+00	1.3594e−01	4.9520e−02	1.9063e+00	1.3375e+01
	Std.	9.4016e−03	4.6420e−02	3.1032e−01	3.7607e−02	3.0453e+00	6.1212e−02	2.4015e−02	4.6006e−01	3.0815e+00
	Rank	1	3	6	4	8	5	2	7	9
$f_{8}$	Mean	-2.5129e+04	-8.4968e+03	-3.5710e+03	-2.3523e+03	6.5535e+04	-8.0867e+03	-3.9428e+03	-3.6621e+03	-6.3312e+03
	Std.	9.5538e+02	7.2587e+02	4.3080e+02	3.8217e+02	0.0000e+00	1.5535e+02	4.0416e+02	2.1416e+02	3.3257e+02
	Rank	1	2	7	8	9	3	5	6	4
$f_{9}$	Mean	0.0000e+00	8.4600e+01	1.2430e+02	3.1000e+01	9.6215e+01	9.2692e+01	1.5284e+02	2.1490e+02	2.3683e+02
	Std.	0.0000e+00	1.6167e+01	1.4251e+01	1.3661e+01	1.9588e+01	1.4224e+01	1.8554e+01	1.7219e+01	1.9034e+01
	Rank	1	3	6	2	5	4	7	8	9
$f_{10}$	Mean	6.9604e−14	1.2604e+00	9.1679e+00	3.7410e+00	1.5946e+01	6.8448e+00	1.9133e+01	1.4568e+01	1.7846e+01
	Std.	1.7583e−13	7.2956e−01	1.5690e+00	1.7127e−01	7.7495e−01	1.2500e+00	2.3852e−01	4.6751e−01	5.3115e−01
	Rank	1	2	5	3	7	4	9	6	8
$f_{11}$	Mean	0.0000e+00	1.9080e−02	1.2419e+01	4.8683e−01	2.2028e+02	2.7161e+00	4.2053e+02	6.9658e+01	1.7990e+02
	Std.	0.0000e+00	2.1732e−02	4.1658e+00	4.9785e−02	5.4707e+01	7.2772e−01	2.5256e+01	1.2114e+01	3.2440e+01
	Rank	1	2	5	3	8	4	9	6	7
$f_{12}$	Mean	8.1573e+03	8.9401e−01	1.3874e+01	4.6344e−01	2.8934e+07	4.1053e+00	8.7428e+06	3.6840e+05	3.4132e+07
	Std.	4.4671e+04	8.8127e−01	5.8537e+00	1.3760e−01	2.1787e+06	1.0435e+00	1.4057e+06	1.7213e+05	1.8934e+06
	Rank	5	2	4	1	8	3	7	6	9
Average		1.5000	2.7500	4.5833	4.9167	7.3333	3.5833	6.4167	6.2500	7.6667
Overall		1	2	4	5	8	3	7	6	9

For deciding whether the results of the IP algorithm generate statistical difference in favor of the IP algorithm compared to the MFO, PSO, GSA, BA, FPA, SMS, FA and GA, the Wilcoxon signed-rank test with the significance level 0.05 is used and test outputs are given in the Table 9. As seen from the test results in the Table 9, the capability of the IP algorithm is also proven statistically. While the IP algorithm is found statistically significant compared to the MFO, PSO, GSA, FPA, SMS and FA with the $\rho $ value equal to 0.00781, its dominance is more apparent compared to the BA and GA with the $\rho $ value equal to 0.00024.

**TABLE 8:** Comparison Between IP Algorithm and Other Meta-Heuristics for 200 Dimensional Problems

Fn.		IPA	ALO [19]	PSO [11], [19]	SMS [19], [38]	BA [14], [19]	FPA [15], [19]	CS [12], [19]	FA [13], [19]	GA [6], [19]
$f_{1}$	Mean	1.1523e−189	7.8900e−07	2.3799e+01	1.0392e+03	1.1173e+03	5.5989e+01	3.8000e−05	7.6128e+01	2.2775e+02
	Std.	0.0000e+00	1.1000e−07	1.1721e+01	4.2430e−01	2.0731e+04	3.2678e+01	1.8500e−05	1.5744e+00	1.8656e+02
	Rank	1	2	4	8	9	5	3	6	7
$f_{2}$	Mean	2.0701e−144	5.3082e+02	2.3787e+02	1.8324e+03	3.8428e+03	2.8060e+02	4.0010e+02	6.1119e+02	6.3226e+03
	Std.	1.1305e−143	2.2267e+02	2.2432e+01	1.2200e−02	4.6828e+02	6.9384e+00	8.6560e−01	7.1219e+01	1.0927e+03
	Rank	1	5	2	7	8	3	4	6	9
$f_{3}$	Mean	1.6826e+05	2.3314e+03	4.6933e+03	2.0349e+03	1.0908e+03	2.4219e+04	1.2957e+04	1.4852e+04	1.1206e+04
	Std.	1.3627e+05	5.0718e+02	5.0357e+02	3.7800e−01	4.7506e+02	8.5400e+03	6.3375e+02	6.4184e+03	3.9861e+03
	9	3	4	2	1	8	6	7	5
$f_{4}$	Mean	8.3428e+01	3.0580e+01	4.0111e+01	3.0026e+02	6.5667e+01	3.7689e+01	3.0936e+01	2.7360e+00	1.0154e+02
	Std.	1.2702e+00	1.1446e+00	5.8790e−01	2.3000e−03	2.8293e+00	2.4572e+00	1.6877e+00	5.4729e−01	2.5321e+00
	Rank	8	3	6	2	7	5	4	1	9
$f_{5}$	Mean	1.9816e+02	1.6704e+02	9.1123e+02	3.8635e+03	1.4108e+03	3.1507e+03	3.3267e+02	1.3217e+03	9.6449e+02
	Std.	3.9420e−01	4.9746e+01	9.5245e+01	5.3290e−01	5.9107e+02	1.4906e+03	1.5988e+02	1.1476e+02	7.4876e+02
	Rank	2	1	4	9	7	8	3	6	5
$f_{6}$	Mean	0.0000e+00	7.6000e−07	4.3421e+01	2.4944e+03	5.1206e+01	1.6699e+02	8.1700e−05	7.8420e+01	4.8256e+02
	Std.	0.0000e+00	7.3900e−08	1.4206e+01	3.0000e−04	1.2005e+01	4.1109e+01	4.5500e−05	2.3405e+00	2.7861e+02
	Rank	1	2	4	9	5	7	3	6	8
$f_{7}$	Mean	7.7848e−04	5.0546e−02	1.7321e+01	2.8359e+01	2.4344e+00	4.8391e+00	4.0131e−01	2.7300e−02	1.1656e+02
	Std.	1.1574e−03	1.4407e−02	4.0133e+00	1.9900e−05	1.2756e−01	1.5354e+00	8.7070e−03	4.1100e−03	6.0161e+01
	Rank	1	3	7	8	5	6	4	2	9
$f_{8}$	Mean	-6.1749e+04	-4.4426e+04	-1.8136e+04	-3.5969e+04	-2.5632e+04	-4.5771e+04	-5.2600e+04	-3.9753e+04	-2.8660e+04
	Std.	4.8328e+02	1.4425e+03	4.9624e+03	8.7650e−01	8.6947e+02	3.0978e+03	1.5604e+02	6.4969e+02	1.0110e+03
	Rank	1	4	9	6	8	3	2	5	7
$f_{9}$	Mean	1.1293e+02	6.1389e+02	7.4858e+02	4.8001e+02	7.2338e+02	7.0295e+02	5.4158e+02	4.7545e+02	1.6458e+03
	Std.	4.6876e+01	6.6795e+01	2.4301e+01	2.3650e−01	1.0096e+02	6.9653e+01	4.1889e+01	2.8058e+01	3.7155e+01
	Rank	1	5	8	3	7	6	4	2	9
$f_{10}$	Mean	4.4409e−16	2.3058e+00	1.5183e+01	1.7293e+01	1.8159e+01	1.7544e+01	1.7654e+01	2.4297e+00	2.0361e+01
	Std.	1.0029e−31	2.5542e−01	5.7627e−01	9.7400e−02	6.7775e−02	1.6684e−01	2.9820e+00	3.8545e−02	1.4256e−01
	Rank	1	2	4	5	8	6	7	3	9
$f_{11}$	Mean	0.0000e+00	7.4240e−03	3.2412e+03	4.8015e+03	4.9370e+03	1.8074e+02	1.1910e−03	1.7048e+00	3.3068e+03
	Std.	0.0000e+00	6.5100e−03	1.3749e+02	8.5320e−01	2.6842e+02	3.6084e+01	1.1480e−03	1.4301e−02	1.1330e+02
	Rank	1	3	6	8	9	5	2	4	7
$f_{12}$	Mean	1.4009e−01	5.3982e+00	4.0700e+05	1.0000e+08	1.6900e+09	4.3700e+07	1.0000e+10	2.3426e+01	8.1400e+09
	Std.	9.6674e−02	5.9591e−01	4.7700e+05	1.9900e−05	4.2800e+08	3.2200e+07	4.5000e−03	5.5985e−01	9.5400e+08
	Rank	1	2	4	6	7	5	9	3	8
Average		2.3333	2.9167	5.1667	6.0833	6.7500	5.5833	4.2500	4.2500	7.6667
Overall		1	2	5	7	8	6	3	3	9

**TABLE 9:** Statistical Comparison Between Meta-Heuristics for 100 Dimensional Problems

IPA vs.	Sum of Ranks		$Z$-val.	$\rho $-val.	Sign.
	$W+$	$W-$			
MFO	0	28	2.41755	0.00781	IPA
PSO	0	28	2.41755	0.00781	IPA
GSA	0	28	2.41755	0.00781	IPA
BA	0	78	3.48710	0.00024	IPA
FPA	0	28	2.41755	0.00781	IPA
SMS	0	28	2.41755	0.00781	IPA
FA	0	28	2.41755	0.00781	IPA
GA	0	78	3.48710	0.00024	IPA

The benchmark functions ranging from $f_{1}$ to $f_{12}$ are solved one more again with IP algorithm by setting the number of parameters to 200 and obtained results of the IP algorithm are compared with the ALO [19], PSO [11], SMS [38], BA [14], FPA [15], CS [12], FA [13] and GA [6]. For a fair comparison between algorithms, the population size is set to 100 and total evaluation number is 500,000 [19]. Each benchmark problem is solved 30 different times with the random seeds and mean best objective function values and standard deviations are presented at the Table 8. The results given in the Table 8 prove that the IP algorithm shows the best performance among the ALO, PSO, SMS, BA, FPA, CS, FA and GA with the average rank calculated as 2.333. While the IP algorithm outperforms other competitors for the $f_{1}$, $f_{2}$, $f_{6}$, $f_{7}$, $f_{8}$, $f_{10}$, $f_{11}$ and $f_{12}$ functions, it lags slightly behind the ALO and becomes the second best algorithm for the $f_{5}$ function. However, when the performance of the IP algorithm on solving $f3$ and $f_{4}$ functions is investigated, it is seen that the unique operations of infection distribution, receiver and donor determination, plasma transfer and subsequent donor modification lose some advantageous sides.

### C. Solving CEC2015 Benchmark Problems With IP Algorithm

In the final part of the experimental studies, the performance of the IP algorithm is analyzed by solving ten different computationally expensive benchmark functions represented at the CEC 2015 [51]. The complexities of the benchmark problems of the CEC 2015 are tried to be increased by applying some rotation, shifting or both rotation and shifting operations. Moreover, some of the problems are generated by combining or hybridizing classical benchmark functions. The lower and upper bounds of the benchmark functions are determined −100 and +100, respectively [51]. The details about the benchmark problems including their names, their base functions and the reference values using as the global minimums are summarized in the Table 10 [51].

**TABLE 10:** CEC 2015 Benchmark Functions Used in the Experiments

Function	Name	Related Basic Functions	Ref.
$f_{1}$	Rotated Bent Cigar Function	Bent Cigar function	100
$f_{2}$	Rotated Discus Function	Discus function	200
$f_{3}$	Shifted and Rotated Weierstrass function	Weierstrass function	300
$f_{4}$	Shifted and Rotated Katsuura function	Katsuura function	500
$f_{5}$	Shifted and Rotated HappyCat function	HappyCat function	600
$f_{6}$	Shifted and Rotated HGBat function	HGBat function	700
$f_{7}$	Shifted and Rotated Expanded Griewank’s plus Rosenbrock’s function	Griewank’s function Rosenbrock’s function	800
$f_{8}$	Shifted and Rotated Expanded Scaffer’s $f_{6}$ function	Expanded Scaffer’s $f_{6}$ function	900
$f_{9}$	Hybrid function 2 (N=4)	Griewank’s function Weierstrass function Rosenbrock’s function Scaffer’s $f_{6}$ function	1100
$f_{10}$	Compositional function 2 (N=3)	Schwefel’s function Rastrigin’s function High conditioned elliptic	1400

Each 30-dimensional benchmark function given in the Table 10 is solved with the IP algorithm for which $NoR$ and $NoD$ parameters are set to 1 and its results are compared with the SOA [31], SHO [30], GWO [17], PSO [11], MFO [18], MVO [23], SCA [21], GSA [34], GA [6] and DE [7] algorithms. For providing a fair comparison, the population size of the algorithms is 100 and total evaluation number is 100,000 for each benchmark function [31]. When the mean best objective function values and standard deviations calculated after 30 different runs in the Table 11 are controlled, it is seen that the IP algorithm with the average and overall ranks equal to 1.8000 and 1 respectively outperforms SOA, SHO, GWO, PSO, MFO, MVO, SCA, GSA, GA and DE. While the $f_{3}$, $f4$, $f_{5}$, $f_{6}$, $f_{7}$, $f_{8}$, $f_{9}$ and $f_{10}$ functions are solved more robustly by the IP algorithm, SOA performs better than the tested meta-heuristics on the $f_{1}$ function and GSA performs better than the tested meta-heuristics on the $f_{2}$ function.

**TABLE 11:** Comparison Between IP Algorithm and Other Meta-Heuristics for 30 Dimensional CEC 2015 Problems

Fn.		IPA	SOA [31]	SHO [30], [31]	GWO [17], [31]	PSO [11], [31]	MFO [18], [31]	MVO [23], [31]	SCA [21], [31]	GSA [31], [34]	GA [6], [31]	DE [7], [31]
$f_{1}$	Mean	1.28e+06	2.55e+05	2.28e+06	2.02e+06	4.37e+05	1.47e+06	6.06e+05	7.65e+06	3.20e+07	8.89e+06	6.09e+06
	Std.	4.46e+05	2.45e+05	2.18e+06	2.01e+06	1.81e+05	1.00e+06	5.02e+05	3.07e+06	2.98e+06	6.95e+06	5.11e+06
	Rank	4	1	7	6	2	5	3	9	11	10	8
$f_{2}$	Mean	5.46e+04	5.53e+06	3.13e+05	5.65e+06	9.41e+03	1.97e+04	1.43e+04	7.33e+08	4.58e+03	2.97e+05	4.40e+04
	Std.	1.01e+04	8.37e+04	2.10e+05	2.19e+06	4.82e+03	1.46e+04	1.03e+04	2.33e+08	1.09e+03	2.85e+03	2.75e+04
	Rank	6	9	8	10	2	4	3	11	1	7	5
$f_{3}$	Mean	3.20e+02	3.20e+02	3.20e+02	3.20e+02	3.20e+02	3.20e+02	3.20e+02	3.20e+02	3.20e+02	3.20e+02	3.20e+02
	Std.	7.21e−03	3.71e−03	3.76e−02	7.08e−02	8.61e−02	9.14e−02	3.19e−02	7.53e−02	1.02e−03	2.78e−02	1.15e−03
	Rank	1	1	1	1	1	1	1	1	1	1	1
$f_{4}$	Mean	5.01e+02	9.54e+02	9.13e+02	9.20e+02	8.65e+02	1.33e+03	1.09e+03	1.76e+03	1.75e+03	1.26e+03	3.34e+03
	Std.	1.64e−01	2.12e+02	1.85e+02	1.78e+02	2.16e+02	3.45e+02	2.81e+02	2.30e+02	2.79e+02	1.86e+02	3.01e+02
	Rank	1	5	3	4	2	8	6	10	9	7	11
$f_{5}$	Mean	6.00e+02	2.47e+03	1.29e+04	2.26e+04	1.86e+03	7.35e+03	3.82e+03	2.30e+04	3.91e+06	2.91e+05	5.39e+04
	Std.	1.91e−02	1.40e+03	1.15e+04	2.07e+04	1.28e+03	3.82e+03	2.44e+03	1.91e+04	2.70e+06	1.67e+05	2.40e+04
	Rank	1	3	6	8	2	5	4	7	11	10	9
$f_{6}$	Mean	7.00e+02	7.02e+02	7.02e+02	7.02e+02	7.02e+02	7.02e+02	7.02e+02	7.06e+02	7.08e+02	7.08e+02	7.06e+02
	Std.	2.71e−02	5.76e−01	6.76e−01	7.07e−01	7.75e−01	1.10e+00	9.40e−01	9.07e−01	1.32e+00	2.97e+00	3.87e+00
	Rank	1	2	2	2	2	2	2	8	11	11	8
$f_{7}$	Mean	8.27e+02	1.65e+03	1.86e+03	3.49e+03	3.43e+03	9.93e+03	2.58e+03	6.73e+03	6.07e+05	5.79e+04	5.60e+03
	Std.	3.86e+00	2.12e+03	1.98e+03	2.04e+03	2.77e+03	8.74e+03	1.61e+03	3.36e+03	4.81e+05	2.76e+04	5.15e+04
	Rank	1	2	3	6	5	9	4	8	11	10	7
$f_{8}$	Mean	9.12e+02	1.00e+03	1.00e+03	1.00e+03	1.00e+03	1.00e+03	1.00e+03	1.00e+03	1.00e+03	1.00e+03	1.00e+03
	Std.	2.10e−01	7.35e−01	1.43e−01	1.28e−01	7.23e−02	2.20e−01	5.29e−02	9.79e−01	5.33e+00	3.97e+00	4.05e+00
	Rank	1	2	2	2	2	2	2	2	2	2	2
$f_{9}$	Mean	1.12e+03	1.32e+03	1.38e+03	1.40e+03	1.35e+03	1.37e+03	1.39e+03	1.35e+03	1.41e+03	1.36e+03	1.65e+03
	Std.	1.29e+00	1.34e+01	2.42e+01	5.81e+01	1.12e+02	8.97e+01	5.42e+01	1.11e+02	7.73e+01	5.39e+01	3.00e+01
	Rank	1	2	7	9	3	6	8	3	10	5	11
$f_{10}$	Mean	1.62e+03	3.78e+03	4.25e+03	7.29e+03	7.10e+03	7.60e+03	7.34e+03	7.51e+03	9.30e+03	8.96e+03	6.08e+03
	Std.	4.09e+00	2.32e+03	1.73e+03	2.45e+03	3.12e+03	1.29e+03	2.47e+03	1.52e+03	1.94e+03	6.32e+03	5.83e+03
	Rank	1	2	3	6	5	9	7	8	11	10	4
Average		1.8000	2.9000	4.2000	5.4000	2.6000	5.1000	4.0000	6.7000	7.8000	7.3000	6.6000
Overall		1	3	5	7	2	6	4	9	11	10	8

In order to investigate that whether the results of the IP algorithm are enough to generate a statistical difference in favor of the same algorithm or not, the Wilcoxon signed-rank test with the significance level equal to 0.05 is utilized and test outputs are listed in the Table 12. While the IP algorithm proves its statistical significance with the $\rho $ values calculated as 0.00195, 0.02734, 0.03710 and 0.04882 compared to SOA, SHO, GWO, MFO, SCA, GSA, GA and DE, its results are not enough to generate statistical difference compared to PSO and MVO algorithms. Even though the the statistical significance is not in favor of IP algorithm compared to PSO and MVO algorithms, it should be noticed that IP algorithm produces better results than both of the PSO and MVO on eight of ten benchmark problems.

**TABLE 12:** Statistical Comparison Between Meta-Heuristics for CEC2015 Problems

IPA vs.	Sum of Ranks		$Z$-val.	$\rho $-val.	Sign.
	W+	W−			
SOA	8	37	1.65632	0.04882	IPA
SHO	0	45	2.88563	0.00195	IPA
GWO	0	45	2.88563	0.00195	IPA
PSO	17	28	0.56759	0.28515	–
MFO	8	37	1.65632	0.04882	IPA
MVO	17	28	0.56759	0.28515	–
SCA	0	45	2.88563	0.00195	IPA
GSA	6	39	1.92135	0.02734	IPA
GA	0	45	2.88563	0.00195	IPA
DE	7	38	1.78526	0.03710	IPA

### D. Solving Signal Decomposition Problems with IP Algorithm

Abbass *et al.* [52] and Goh *et al.* [53], [54] have recently introduced a new optimization problem that has a strong relationship with the big data concept of the computer science and the unique properties of the newly introduced problem have attracted the researchers for solving it with the techniques developed by guiding the well-known meta-heuristics. They directly utilized from the measurement results of the EEG signals when describing their problems and stated that decomposing the obtained EEG signals into two different parts is the main motivation of the problem. The EEG measurements for a second are classified based on the number of time series and problem instances with four time series are named as D4 and D4N and problem instances with twelve time series are named as D12 and D12N [52]–[54]. In the D4 and D4N, four signal sources are utilized for measurements and two signal sources are used in modeling of the noise part [52]–[54]. In the other problem instances, twenty-five different signal sources are used for both obtaining required EEG signal and measurement artifacts. While the D12 and D12N instances are related with the first twelve signal sources, the remaining signal sources are overlapped with the signal sources used for obtaining main EEG signals. Even though measurement noise is included in all problem instances, the problem instances ended with ${N}$ abbreviation are also complicated with the extra noise addition as their names imply [52]–[54].

Given that $X$ is a matrix and it has $N$ rows and $M$ columns. The $N$ value actually represents the number of time series and $M$ value corresponds to the lengths of each time series. $S$ is an $N \times M$ dimensional matrix and it represents one of the four problem instances. Also, $A$ is a square matrix of size $N \times N$ and used to transform $S$ into $X$ matrix. For understanding how the $X$, $S$ and $A$ matrices are related with each other, the Eq. (6) should be controlled [52]–[54]. As stated earlier, the main purpose of the problem is decomposing the $S$ matrix into appropriate matrices. The noiseless component of the $S$ matrix is matched with the $S1$ matrix and it is known that the $S1$ should be similar to the original $S$ matrix. In addition to this, the noise or measurement artifacts on the $S$ matrix corresponds to the $S2$ matrix [52]–[54]. Although the $S$ matrix is decomposed into $S1$ and $S2$ matrices, the sum of $S1$ and $S2$ matrices must be equal to the $S$ matrix and the sum of transformed $S1$ and $S2$ matrices must also be equal to the $X$ matrix as described in the Eq. (7) and Eq. (8), respectively [52]–[54].

Although the connection of these matrices is described with simple matrix operations, obtaining the $S1$ and $S2$ matrices from $S$ by considering described purpose of the problem is not so straightforward. However, Abbass *et al.* [52] and Goh *et al.* [53], [54] showed that some statistical indicators about the $X$, $A$ and $S1$ matrix being guessed can be useful for guiding the division of the $S$ matrix. One of the statistical indicators that can be utilized for guiding the division of the $S$ matrix and calculated as in Eq. (9) is related with the correlation coefficients of Pearson [52]–[54]. In the Eq. (9), $C$ is the matrix showing Pearson correlation coefficients and $var(X)$ is the variance matrix. Also, $var(A \times S1)$ and $covar(X,A \times S1)$ are variance and covariance matrices, respectively [52]–[54]. While the $S1$ is extracted from the original $S$ matrix, it is considered that the off-diagonal elements of the $C$ matrix should be minimized and the other elements of the same $C$ matrix should be maximized [52]–[54].

\begin{align*} X=&A \times S \tag{6}\\ S=&S1 + S2 \tag{7}\\ X=&A \times S1 + A \times S2 \tag{8}\\ C=&\frac {covar(X, A \times S1)}{var(X) \times var(A \times S1)} \tag{9}\end{align*}

After splitting $S$ matrix into $S1$ and $S2$ matrices and calculating $C$, the relevance of the $S1$ matrix with the original $S$ matrix and appropriateness of the $C$ matrix should be measured. By aiming at determining quality of the division, Abbass *et al.* [52] and Goh *et al.* [53], [54] presented objective functions given in the Eq. (10) and Eq. (11). The objective function given in the Eq. (10) describes the appropriateness of the matrix showed by $C$. Other objective given in Eq. (11) is devoted to the calculation of similarity between the $S$ and $S1$ matrices [52]–[54]. When the first and second objective function values are investigated, it is understood that an accurate signal decomposition should minimize them. If the $S1$ matrix that minimizes the sum of the equally weighted $f_{1}$ and $f_{2}$ given as $(f_{1} + f_{2})$ is tried to be found, an optimization problem with the single objective is easily introduced [52]–[54]. If both $f_{1}$ and $f_{2}$ objective functions are tried to be minimized simultaneously, it is said that there are more than one objectives required to be optimized and a multi-objective big data optimization problem can be defined. For single and multi-objective interpretations of the problems, lower and upper bounds of the elements in $S1$ matrix are set to −8 and +8 [52]–[54].

\begin{align*} f_{1}=&\frac {1}{(N^{2} - N)}\sum _{i \neq j}{}(C_{ij})^{2} + \frac {1}{N}\sum _{i}{}(1-C_{ii})^{2} \tag{10}\\ f_{2}=&\frac {1}{N \times M}\sum _{ij}{}(S_{ij}-S1_{ij})^{2} \tag{11}\end{align*}

In order to analyze that whether the IP algorithm still protects its capabilities for complex engineering problems or not, single objective D4, D4N, D12 and D12N instances are solved with the IP algorithm and its results are compared with the results of the GA [6], PSO [11], DE [7], ABC [16], GSA [34], MFO [18], SCA [21] and SSA [25]. For each algorithm, the population or colony size is set to 50 and maximum fitness evaluations are taken equal to 10,000. The crossover and mutation probabilities of the GA is 0.95 and 0.001, respectively [6]. In addition to these, the best 20% of the whole GA population are preserved at each generation by the elitism strategy. For the PSO algorithm, given that $x_{j}^{min}$ and $x_{j}^{max}$ are lower and upper bounds of the $jth$ decision parameter, lower and upper bounds of the velocity are $(x_{j}^{min}-x_{j}^{max}) \times 0.2$ and $(x_{j}^{max}-x_{j}^{min}) \times 0.2$ [11]. While the inertia weight is determined between 0.2 and 0.9, the $c_{1}$ and $c_{2}$ acceleration coefficients are taken equal to 2 [11]. The crossover rate of the DE is set to 0.90 and the scaling factor is adjusted randomly between 0.2 and 0.8 [7]. The *limit* parameter of the ABC algorithm is calculated as $(PS \times D)/2$ [16]. For the MFO, the $b$ constant used in the calculations of the logarithmic spiral is 1 [18]. The $a$ constant of the SCA is set to 2 [21]. The $c_{1}$ coefficient of the SSA is calculated by using the formula $2e^{-(16l^{2}/L^{2})}$ where $l$ is the current iteration and $L$ is the maximum number of iterations [25]. Finally, the $NoR$ and $NoD$ parameters of the IP algorithm are set to 8 and 4.

For each algorithm, problem instances are solved 30 times with different random seeds. The mean best, best objective function values and standard deviations are recorded and presented at the Table 13. The results given in the Table 13 show that the IP algorithm filters the measurement noise and obtains more robust EEG signals compared to the GA, PSO, DE, ABC, GSA, MFO, SCA and SSA algorithms for all of the four problem instances. While the mean best objective function values obtained by the IP algorithm are between 1.0597 and 12.3272 times better than its competitors for the D4 instance, the mean best objective function values obtained by the IP algorithm are also between 1.0353 and 11.9848 times better than its competitors for the D4N instance. Moreover, while the mean best objective function values obtained by the IP algorithm are between 1.1955 and 12.1916 times better than its competitors for the D12 instance, the mean best objective function values obtained by the IP algorithm are also between 1.2041 and 12.2223 times better than its competitors for the D12N instance. The effect of the plasma that is extracted from a donor and transferred into a receiver becomes more dominant on the exploitation mechanism of the IP algorithm and significantly improves the qualities of the final solutions when the difficulty and dimensionality of the problems increase as in the considered signal decomposition problems. Modifying a receiver solution by changing all of the parameters with the support of the donor or the plasma extracted from it helps preserving promising solution more longer in the population and searching the vicinity of the promising solution more steadily.

**TABLE 13:** Comparison Between IP Algorithm and Other Meta-Heuristics for D4, D4N, D12 and D12N Instances

Alg.		Instances			
		D4	D4N	D12	D12N
IPA	Mean	1.6599e+00	1.6989e+00	1.8370e+00	1.8359e+00
	Best	1.5458e+00	1.5871e+00	1.7710e+00	1.7076e+00
	Std.	6.1728e−02	5.2602e−02	3.8295e−02	5.2990e−02
	Rank	1	1	1	1
GA	Mean	2.1300e+00	2.1491e+00	2.8016e+00	2.7769e+00
	Best	1.8930e+00	1.8715e+00	2.5454e+00	2.3848e+00
	Std.	1.5558e−01	1.5647e−01	1.5078e−01	1.5087e−01
	Rank	3	3	3	3
PSO	Mean	7.8788e+00	7.8962e+00	1.0634e+01	1.0613e+01
	Best	7.2433e+00	7.5527e+00	1.0024e+01	1.0271e+01
	Std.	2.7104e−01	2.2522e−01	1.9250e−01	1.5696e−01
	Rank	6	6	6	6
DE	Mean	1.4654e+01	1.6886e+01	2.2396e+01	2.2439e+01
	Best	7.4238e+00	5.3421e+00	2.1890e+01	2.2020e+01
	Std.	6.3289e+00	7.0483e+00	2.0155e−01	1.7023e−01
	Rank	7	7	9	9
ABC	Mean	2.0042e+01	2.0101e+01	2.1958e+01	2.1954e+01
	Best	1.8982e+01	1.9608e+01	2.1599e+01	2.1431e+01
	Std.	4.2809e−01	3.2692e−01	1.9688e−01	2.2401e−01
	Rank	8	8	7	7
GSA	Mean	1.7590e+00	1.7588e+00	2.1962e+00	2.2106e+00
	Best	1.6384e+00	1.5660e+00	2.0587e+00	2.1154e+00
	Std.	7.9340e−02	8.6096e−02	5.6204e−02	4.3377e−02
	Rank	2	2	2	2
MFO	Mean	2.0462e+01	2.0361e+01	2.2027e+01	2.1970e+01
	Best	1.9546e+01	1.9852e+01	2.1819e+01	2.1394e+01
	Std.	3.1975e−01	3.5109e−01	1.2472e−01	2.2827e−01
	Rank	9	9	8	8
SCA	Mean	6.0747e+00	6.4380e+00	6.9154e+00	6.8059e+00
	Best	3.0410e+00	3.5316e+00	5.8082e+00	2.3134e+00
	Std.	1.4271e+00	1.0361e+00	4.6994e−01	1.0449e+00
	Rank	5	5	5	5
SSA	Mean	2.7771e+00	2.8256e+00	3.0998e+00	3.1211e+00
	Best	2.6027e+00	2.6706e+00	2.9668e+00	2.9541e+00
	Std.	1.1751e−01	8.5527e−02	7.3073e−02	7.7361e−02
	Rank	4	4	4	4

## IV. Conclusion

The convalescent plasma or immune plasma is one of the well-known treatment methods based on transferring the antibodies of an individual who has recovered previously to another patient or patients of the same infection. In this study, the simple but efficient idea lying behind the plasma treatment was investigated and a new meta-heuristic algorithm named as Immune Plasma algorithm for short IPA was introduced. Each individual of a population in IP algorithm is matched with the possible solution of the optimization problem being solved and immune response of an individual to the spreading infection corresponds to the appropriateness or quality of the solution. In order to analyze the capabilities of the IP algorithm, a set of experimental studies was conducted. In the first part of the experiments, thirteen classical benchmark problems were solved with IP algorithm by assigning different values to the specific $NoR$ and $NoD$ parameters. The second part of the experimental studies was devoted to the analysis the IP algorithm on high dimensional benchmark problems. In the third part of the experimental studies, complex benchmark problems presented at the CEC 2015 were solved with the IP algorithm. Finally, in the fourth part of the experimental studies, the IP algorithm was used to solve a complex engineering problem.

The results of the experimental studies were compared with the standard implementations of the previously introduced well-known meta-heuristic algorithms including GA, PSO, DE, RCBBO, CS, FA, GSA, AMO, ABC, BA, MFO, FPA, SMS, ALO, SOA, SHO, GWO, MVO, SCA and SSA. From the detailed comparisons between IP algorithm and other meta-heuristics, it was concluded that IP algorithm is capable of obtaining better solutions for most of the benchmark problems. The model used to distribute infection between individuals provides an efficient exploration mechanism. This efficient exploration mechanism is balanced by a detailed exploitation operation in which donor individual or individuals are used as plasma sources for the emerging patient or patients and more than one dose plasma can be transferred to the patient or patients if required. In future, the performance of the IP algorithm can be analyzed by solving different constrained or non-constrained single and multi-objective optimization problems. The discrete variants of the IP algorithm can also be developed and tested. Moreover, IP algorithm can be modified by using different infection distribution approaches or adaptive IP algorithm variants by assigning required values to the $NoR$ and $NoD$ parameters for each infection period dynamically can be introduced.
